# Sea lice (Copepoda: Caligidae) from South Africa, with descriptions of two new species of *Caligus*

**DOI:** 10.1007/s11230-021-09984-2

**Published:** 2021-06-26

**Authors:** Polly M. Hayes, Kevin W. Christison, David B. Vaughan, Nico J. Smit, Geoffrey A. Boxshall

**Affiliations:** 1 Department of Life Sciences, Natural History Museum, Cromwell Road, London, SW7 5BD UK; 2 School of Life Sciences, College of Liberal Arts and Sciences, University of Westminster, London, W1W 6UW UK; 3 Department of Forestry, Fisheries and the Environment, Private Bag X2, Vlaeberg, 8012 South Africa; 4 Department of Biodiversity and Conservation Biology, University of the Western Cape, Private Bag X17, Bellville, 7535 South Africa; 5 Central Queensland University, School of Access Education and Coastal Marine Ecosystems Research Centre, Rockhampton, QLD 4701 Australia; 6 Water Research Group, Unit for Environmental Sciences and Management, North-West University, Potchefstroom, 2520 South Africa

**Keywords:** parasitic copepods, fish hosts, taxonomy, marine fish parasites

## Abstract

Thirteen species of sea lice (family Caligidae) are reported from a range of elasmobranch and actinopterygian fishes caught off South Africa or obtained from public aquaria in South Africa. Two new species of *Caligus* Müller, 1785 are described: *C. linearis* **n. sp.** from *Pomatomus saltatrix* (Linnaeus) and *C. tumulus* **n. sp.** from *Chrysoblephus cristiceps* (Valenciennes). A supplementary description is provided for both sexes of *Caligus tetrodontis* Barnard, 1948 taken from *Amblyrhynchotes honckenii* (Bloch) and previous records of this parasite from South African fishes are critically reviewed. It is concluded that *Caligus* material from *Arothron hispidus* Linnaeus was previously misidentified as *C. tetrodontis* and is in urgent need of re-examination. Morphological and molecular observations on *Caligus furcisetifer* Redkar, Rangnekar & Murti, 1949 indicate that this copepod is phenotypically and genetically identical to *Lepeophtheirus natalensis* Kensley & Grindley, 1973, and the latter becomes a junior subjective synonym of *C. furcisetifer*. We include new geographical distribution records for *Caligus longipedis* Bassett-Smith, 1898, *C. rufimaculatus* Wilson, 1905 and *Lepeophtheirus spinifer* Kirtisinghe, 1937, extending into South African waters, as well as both new distribution and host records for *Alebion gracilis* Wilson, 1905, *Caligus dakari* van Beneden, 1892 and *Lepeophtheirus acutus* Heegaard, 1943. The molecular analysis confirmed the monophyly of the genus *Caligus.* The South African species of *Caligus* did not cluster together, but the two included South African species of *Lepeophtheirus* were recovered as sister taxa.

## Introduction

The family Caligidae Burmeister, 1835 currently comprises 513 valid species in 31 genera (Walter & Boxshall, 2020) and over half of these species (270 species) belong to the genus *Caligus* Müller, 1785 (Boxshall & Hayes, 2019). This genus has a pan-global distribution and is known from a broad range of fish hosts, predominantly teleosts. Only nine genera of the family Caligidae are currently known from southern African marine fishes (Dippenaar, 2005) including four genera formerly belonging to the Family Euryphoridae which has been synonymised with the family Caligidae (see Boxshall & Halsey, 2004; Dojiri & Ho, 2013). These nine genera are: *Alebion* Krøyer, 1863 (five species), *Caligodes* Heller, 1865 (one species), *Caligus* Müller, 1785 (38 species), *Euryphorus* Milne Edwards, 1840 (two species), *Gloiopotes* Steenstrup & Lütken, 1861 (one species), *Hermilius* Heller, 1865 (two species), *Lepeophtheirus* von Nordmann, 1832 (nine species), *Paralebion* Wilson, 1911 (one species), and *Tuxophorus* Wilson, 1908 (one species) (Dippenaar, 2005; 2018). The genus *Pseudocaligus* Scott, 1901 was regarded as valid in 2004 (Boxshall & Halsey, 2004; Dippenaar, 2005) but has since been synonymised with *Caligus* (Dojiri & Ho, 2013; Freeman et al., 2013; Özak et al., 2013). The single species of *Pseudocaligus* reported from southern Africa*, P. apodus* Brian, 1924, is included in the above list as *Caligus apodus*. The validity of the genus *Sciaenophilus* van Beneden, 1852 has been questioned repeatedly (see Kabata, 1979) but was accepted by Dojiri & Ho (2013). However, it has recently been synonymised with *Caligus* by Özak et al. (2017) so the type species *Sciaenophilus tenuis* van Beneden, 1852 is reported here under the combination *Caligus tenuis* (van Beneden, 1852).

Several species, predominantly from the genera *Lepeophtheirus* and *Caligus,* have emerged as serious pests of finfish in commercial aquaculture facilities globally (Johnson et al., 2004). Fish lice of the family Caligidae typically have direct life-cycles and hence the infection of new susceptible hosts is horizontal from an infected host to other susceptible hosts. Dispersal of these parasites is achieved through non-feeding planktonic nauplii and the free-living, infective planktonic copepodid stage which locates and attaches to a new host. The ectoparasitic stages on the host include two or four chalimus stages, two pre-adults (in species with only two chalimus stages) and the adults (Ohtsuka et al., 2009; Hamre et al., 2013). Fish host mortalities have been associated with severe ectoparasitic caligid infestations in captive fishes through host osmoregulatory failure, anaemia, ulcerations, or through the facilitation of secondary infections (Hutson et al., 2007; Johnson et al., 2019)

This study aims to document representatives of the family Caligidae obtained from wild-caught elasmobranch and actinopterygian hosts collected for use as aquaculture brood stock fish or display fish for public aquaria. These records cover three of the nine genera represented in southern Africa and include *Alebion* (one species)*, Caligus* (nine species) and *Lepeophtheirus* (three species). Initial identifications were based on morphological characters but where possible molecular studies were also undertaken in order to confirm identifications, explore phylogenetic relationships, and provide reference DNA sequences for future use.

## Materials and methods

Specimen collection

The fish intended for use as aquaculture brood stock or exhibit in public aquaria were caught and landed through commercial and recreational fishing activity. The parasites reported in this study were isolated from infected hosts as part of routine health screening and animal welfare procedures for fish held in quarantine. Parasite collection from the fish hosts was non-invasive and non-destructive. Although this use of the fish was not subject to an intervention covered by South African legislation involving the use of animals in scientific procedures, the fish were handled humanely and in accordance with national and organisational regulations.

*Morphological methods.* Prior to morphological examination the specimens were cleared in lactic acid for 2 h, and mounted on glass slides as temporary preparations in lactophenol. Drawings were made using a drawing tube on a Leitz Diaplan microscope with differential interference contrast and measurements were made using an ocular micrometer. Terminology follows Boxshall (1990) and Huys & Boxshall (1991); host fish names are according to FishBase (Froese & Pauly, 2019). Type and voucher specimens are deposited in the collections of the Iziko South African Museum (SAMCTA) and in the Natural History Museum, London (NHMUK).

*DNA extraction.* Additional voucher specimens used for molecular analysis were all fixed and stored in 70-100% ethanol. Total genomic DNA (gDNA) was extracted from specimens (individual representative male/female adults where available) using the DNeasy Blood & Tissue kit (QIAGEN) following the manufacturer’s instructions for animal tissue, with the exception that the proteinase-K incubation step was extended to overnight and the final elution volume was 200 µl. For samples with low gDNA yields the elution volume was reduced to 100 µl using a vacuum centrifuge in order to increase the final gDNA concentration. Prior to gDNA extraction tissue homogenisation was achieved by physical maceration using a sterile teflon pestle and/or sterile scalpel blade.

*PCR amplification and DNA sequencing:* Genetic sequence data were generated for one mitochondrial DNA (mtDNA) region, the partial cytochrome *c* oxidase subunit 1 (CO1) region, and one ribosomal DNA (rDNA) region, the small ribosomal subunit (18S) rDNA coding region. PCRs were carried out in 25 µl reaction volumes using either (a) Dream Taq PCR Master Mix (2X) (Fermentas), or for samples that proved particularly difficult to amplify, (b) Ready-to-go PCR beads (Amersham Biosciences). In the case of (a), reactions comprised of 12.5 µl of DreamTaq PCR Master Mix (2X) (containing Dream Taq DNA Polymerase, optimized DreamTaq buffer, MgCl_2_ and dNTPs), 2-10 µl of gDNA, 2 µl each of the forward and reverse primers, and PCR grade water to a final reaction volume of 25 µl. For (b), 2-10 µl of gDNA and 2 µl each of the forward and reverse primers were added to GE Healthcare ‘Ready-to-go’ PCR beads (Amersham). Final reactions, in both cases, were made up to 25 µl with PCR grade water (Fisher).

The barcode region of the CO1 mtDNA gene was amplified using universal primers LCO4190 (5′-GGTCAACAAATCATAAAGATATTGG-3′) and HCO2198 (5′-TAAACTTCAGGGTGACCAAAAAATCA-3′) (Folmer et al., 1994), using the following cycling conditions: 5 min initial denature at 95°C, followed by 37 cycles of 30 s at 95°C, 30 s at 47°C, 1 min at 72°C; and 7 min final extension at 72°C (modified from Øines & Heuch, 2005).

18S rDNA was amplified using primers F18Scaligus53 (5′-GCCAGTAGTCATATGCT-3′) and R18Scaligus35 (5′-TTGCCCTCCAGAGGTT-3′) (Øines & Schram, 2008), or in overlapping fragments using a combination of primers 18Sf (5′-TACCTGGTTGATCCTGCCAG-3′) and 614r (5′-TCCAACTACGAGCTTTTTAACC -3′), 554f (5′-AAGTCTGGTGCCAGCAGCCGC-3′) and 1282r (5′-TCACTCCACCAACTAAGAACGGC-3′), 1150f(p2) (5′-ATTGACGGAAGGGCACCACCAG-3′) and 18Sr (5′-TAATGATCCTTCCGCAGGTTCAC-3′) (Huys et al., 2007), and 18SpartF (5′-CAGGGTTCGATTCCGGAGAG-3′) and 18SpartR (5′-CCACCAACTAAGAACGGCCA-3′) (this study). Cycling conditions for 18S primers were: 5 min initial denature at 95°C, followed by 37 cycles of 1 min at 95°C, 1 min at 52°C (F18Scaligus53 and R18Scaligus35), 55°C (18Sf and 18Sr; 18SpartF and 18SpartR) and 59°C (554f and 1282r; 1150f(p2) and 18Sr), 2 min at 72°C; and 10 min final extension at 72°C (modified from Huys et al., 2007). New primers used in this study were designed using Primer BLAST (www.ncbi.nlm.nih.gov/tools/primer-blast/).

PCR reactions were performed using a Veriti 96 well thermal cycler (Applied Biosystems^TM^) PCR machine and 5µl of each amplicon was visualised with gel red stain (Bioline) in 1% agarose gels. The remaining PCR products were purified and sequencing of both strands was carried out on an Applied Biosystems 3730 DNA analyser, using the appropriate PCR primers with Fluorescent Dye Terminator Sequencing Kits (Applied Biosystems^TM^).

*DNA sequence alignment and phylogenetic reconstruction.* Resultant CO1 and 18S sequences were assembled and edited manually using Bioedit (Hall, 1999). Sequence identity was checked using the Basic Local Alignment Tool (BLAST) (http://www.ncbi.nih.gov/BLAST/). The two gene sequences for each species were concatenated and then aligned with concatenated published 18S and CO1 sequences of other caligids using the MUSCLE sequence alignment tool (http://www.ebi.ac.uk) and then visualised and edited in BioEdit (Hall, 1999). Genbank sequences utilised in the final phylogenetic analysis are provided in Table 1. The free-living/non-parasitic cyclopoid *Cyclops insignis* (GenBank accession numbers: EF532821, GU055752) was selected as the out-group. Bayesian inference analysis was performed on a concatenated dataset to provide more robust identification and phylogenetic analysis of species using PhyloSuite (Zhang et al., 2020). The analysis was run using the GTR+I+G model as determined by ModelFinder in PhyloSuite. The analysis was run with two independent runs, each with four chain sets (heated chains temp = 0.2) run for 2,000,000 generations and sampled every 1000 generations, with 100,000 generations discarded as ‘burn-in’.

**Table 1:** Caligid species included in the phylogenetic analyses with GenBank accession numbers

**Species**	**GenBank ID**	
	**18S**	**CO1**
*Caligus belones* Krøyer, 1863	EF088405	AY861368
*Caligus brevipedis* Bassett-Smith, 1896	EF088416	KC345610
*Caligus centrodonti* Baird, 1850	EF088406	AY861370
*Caligus clemensi* Parker & Margolis, 1964	DQ123833	HQ157566
*Caligus curtus* Müller, 1785	EF088407	AY861366
*Caligus elongatus* von Nordmann, 1832	EF088408, EF088409	AY386273, AY386272
*Caligus fugu* (Yamaguti, 1936)	KC569364	KC569364
*Caligus gurnardi* Krøyer, 1863	EF099410	AY861369
*Caligus lacustris* Steenstruup & Lütken, 1861	MT937089	MT920724
*Caligus pelamydis* Krøyer, 1863	EF088411	AY861367
*Caligus punctatus* Shiino, 1955	KR048777	KR049057
*Caligus quadratus* Shiino, 1954	EF088412	EF065619
*Caligus rogercresseyi* Boxshall & Bravo, 2000	AY174153	HQ157565
*Caligus uniartus* (Ho.Kim, Cruz & Nagasawa, 2004)	KC569363	KC569367
*Lepeophtheirus chilensis* Wilson, 1950	JX896386	KU317572
*Lepeophtheirus frecuens* Castro-Romero & Baeza-Kuroki, 1984	JX896390	KU317560
*Lepeophtheirus goniistii* Yamaguti, 1936	KR048779	KR049054
*Lepeophtheirus hippoglossi* (Krøyer, 1837)	EF088404	AY861362
*Lepeophtheirus hospitalis* Fraser, 1920	DQ123831	HM800843
*Lepeophtheirus longicauda* (Markevich, 1940)	LC512444	LC512441
*Lepeophtheirus natalensis* Kensley & Grindley, 1973	FJ447440	FJ447375
*Lepeophtheirus parviventris* Wilson, 1905	KR048780	KR049055
*Lepeophtheirus pectoralis* (Müller, 1776)	EF088413	AY861364
*Lepeophtheirus pollachius* Bassett-Smith, 1896	EF088414	AY861363
*Lepeophtheirus salmonis* Krøyer, 1837	AF208263	AY625897
*Lepeophtheirus thompsoni* Baird, 1850	EF088415	EF065617
*Lepeophtheirus yanezi* Stuardo & Fagetti, 1961	JX896402	KU317594

In order to assess the relationship between closely related species, uncorrected pairwise genetic distance (*p*-distance) between selected sequences was calculated as a measure of divergence, using Mega X (Kumar et al., 2018). The standard 3% divergence for DNA barcodes (Herbert et al., 2003) was used as an indication of distinct species, with any measure of divergence below this threshold considered to indicate likely synonyms.

## Results

**Genus:** ***Alebion*** **Krøyer, 1863**

Type species: *Alebion carchariae* Krøyer, 1863, by monotypy.

***Alebion gracilis*** **Wilson, 1905**

*Host*: *Carcharias taurus* (Rafinesque, 1810)

*Locality*: Mgwalana, Eastern Cape, South Africa (33°24′56.58″S, 27°16′38.51″E), collected on 20 November 2006

*Material examined*: 1 female, 1 male and 2 developmental stages. Vouchers: 1 female, 1 male and 1 developmental stage deposited in the collections of the Iziko South African Museum, (SAMC-A088680).

*Description*: Cressey (1972) revised the genus *Alebion* and provided detailed redescriptions of both sexes of *A. gracilis*.

*Remarks*:

A single adult of each sex was present plus two developmental stages, one of which was used for molecular sequencing. Cressey (1972) revised this genus and provided keys to the eight species he accepted as valid. Since that revision *Alebion difficile* (van Beneden, 1892) has been resurrected as valid (Dippenaar, 2018). The female of *A. gracilis* possesses long posterior processes and prominent lateral bulges on the genital complex, and the first abdominal somite has well developed lateral lobes (referred to as alae by Cressey, 1972). The paired spermatophores attached to the ventral surface of the female complex are not divergent and lack sinuses or swellings at the anterior end. In addition, the maxillipeds of the female have a simple claw and the postoral adhesion pad has linear surface markings. This combination of character states would allow the female to be keyed out as *Alebion gracilis*, but the spermatophores appear relatively longer than those figured by Cressey (1972) and there is no “sclerotized ring” on the adjacent body surface. So, the female keys out as *A. gracilis* but exhibits some minor differences from the description presented by Cressey (1972). The modified outer spine on the second exopodal segment of leg 2 of the adult male extends only to about mid-length of the modified spine on the third segment and the markings on the postoral adhesion pad are linear all over its surface. The combination of these two character states allows the male to be keyed out as *A. gracilis*. The material is provisionally identified as *A. gracilis* although the female in particular exhibits some differences from the published description of Cressey (1972). Having only a single female prevents us from assessing the significance of these morphological differences.

According to Cressey (1972) the confirmed distribution of *A. gracilis* was restricted to the east coast of North America, so this first report from South Africa represents a significant extension of its known geographical distribution. *Carcharias taurus* is a new host record for this parasite.

**Genus:** ***Caligus*** **Müller, 1785**

Type species: *Caligus curtus* Müller, 1785, by monotypy.

***Caligus dakari*** **van Beneden, 1892**

*Host*: *Argyrosomus japonicus* (Temminck & Schlegel, 1843)

*Locality*: Witsand, South Africa (34°24′8.19″S, 20°48′59.50″E), collected on 15 November 2002

*Material examined*: 5 females and 1 male. Vouchers: 3 females in the Iziko South African Museum, (SAMC-A088681); 2 females and 1 male in the Natural History Museum (London), (NHMUK 2015.485-487).

*Representative DNA sequences*: GenBank: MW911362, MW925120

*Description*: The most recent description of *C. dakari* is Boxshall & El-Rashidy (2009).

*Remarks*: Van Beneden (1892) briefly described female *C. dakari* from an unknown fish host caught in Dakar Bay, Senegal. It was subsequently reported by Thompson & Scott (1903) from *Arius venosus* Valenciennes caught off Sri Lanka, and by Kirtisinghe (1964) from *Arius* sp., also from Sri Lanka. Kirtisinghe (1964) also considered that the specimens of *C arii* Bassett-Smith, 1898 reported from South Africa by Barnard (1948, 1955) belonged to *C. dakari*. In his monograph on the parasitic copepods on Indian marine fishes, Pillai (1985) was unable to confirm the presence of *C. dakari* in Indian waters as he could not obtain any material to examine. Pillai did, however, include the species in his monograph (Pillai, 1985). *Caligus dakari* from *Plicofollis dussumieri* (Valenciennes) (as *Ariodes dussumieri*) was included in the list of southern African caligids by Dippenaar (2005), based on the record of Barnard (1948) from Chinde, Mozambique.

The revision of the *Caligus productus* group by Boxshall & El-Rashidy (2009) recognized the typical form of *C. mauritanicus* Brian, 1924 as a junior subjective synonym of *C. dakari*. Boxshall & El-Rashidy (2009) accepted only the original description of *C. dakari* from an unknown host caught in Dakar Bay and the record of Brian (1924) from *Lichia amia* (Linnaeus) and *Argyrosomus regius* (as *Sciaena aquila*) as confirmed. They listed the known distribution as the Eastern South Atlantic (Mauritania, Senegal) only. In our opinion, the identity of the *Caligus* species found on ariid catfish in southern Africa requires confirmation. *Caligus arii* is a valid species which possesses 3 plumose setae on the posterior margin of the distal exopod segment of leg 1 (Pillai, 1963), and is therefore not closely related to *C. dakari* which lacks such setae (the lack of these setae is the diagnostic feature of the *C. productus* group).

Although *C. dakari* has previously been recorded from the sciaenid *Argyrosomus regius* (e.g. Brian, 1924), this is the first record of this parasite from its congener *A. japonicus* (Sciaenidae). Given the uncertainty over the identity of the *C. arii* reported by Barnard (1948), this may also represent a range extension south from Mauritania and Senegal, into South African waters. In a published conference abstract, Grobler et al. (2003) reported an unidentified *Caligus* sp. from *Argyrosomus japonicus* from De Hoop Nature Reserve on the southern coast of South African. It is possible this parasite might be *C. dakari* but its identity can only be confirmed after examination of the material.

***Caligus furcisetifer*** **Redkar, Rangnekar & Murti,** 1949

Syn. *Caligus lepeophtheiropsis* Pillai, 1968

*Lepeophtheirus natalensis* Kensley & Grindley, 1973 (new synonym)

*Host*: *Carcharias taurus* (Rafinesque, 1810)

*Locality*: Jeffreys Bay (34° 3′7.34″S, 24°56′0.68″E), collected on 24 March 2006

*Material examined*: 75 females and 3 males. Vouchers: 65 females and 2 males in the Iziko South African Museum, (SAMCTA-A-88682); 10 females and 1 male in the Natural History Museum, London (NHMUK 2015.510-520).

*Representative DNA sequences*: GenBank: MW911361, MW925119

*Description*: *Caligus furcisetifer* was redescribed in detail by Morgan et al. (2010).

*Remarks*: *Caligus furcisetifer* has previously been reported from only two host species, *Pristis microdon* Latham (Morgan et al., 2010) and *Eusphyra blochii* (Cuvier) (Redkar et al., 1949). It was reported from a *Pristis* sp. by Pillai (1968) under the name *Caligus lepeophtheiropsis*, which Pillai (1985) himself subsequently recognized as a synonym of *C. furcisetifer*. New records of *C. furcisetifer* on sawfish, *Pristis microdon*, were recently published by Morgan et al. (2010) which extended its known range from India (Pillai, 1985) to northern Australia. Boxshall (2018) reported *C. furcisetifer* on *Glaucostegus typicus* (Anonymous [Bennett]) in Moreton Bay, Queensland.

*Lepeophtheirus natalensis* was first described by Kensley & Grindley (1973), based on six ovigerous females collected from *Carcharinus leucas* Müller and Henle caught off KwaZulu-Natal, South Africa. It was subsequently reported from *Carcharias taurus* taken by Olivier et al. (2000) off KwaZulu-Natal. Dippenaar (2009) sequenced *L. natalensis* in her molecular based study of six families of siphonostomatoids found on elasmobranch hosts, and posted 18S and COI sequences in GenBank. *Lepeophtheirus natalensis* tends to be recovered separate from other *Lepephtheirus* species in sequence-based analyses of relationships within the Caligidae (e.g. Freeman et al., 2013), and this has fueled doubts concerning the monophyletic status of *Lepeophtheirus* (e.g. Morales-Serna et al., 2014). However, close inspection of the original description of *L. natalensis* reveals multiple fine scale morphological similarities with *Caligus furcisetifer* which is one of very few *Caligus* species to occur on elasmobranch hosts. On the basis of morphology alone, we suspected that *L. natalensis* is a synonym of *C. furcisetifer*, as the only difference between these two taxa is the absence of the lunules present on the frontal plates of *C. furcisetifer*. These lunules are tiny and difficult to see, and we consider that they may have been overlooked by Kensley & Grindley (1973).

We sequenced *C. furcisetifer* from *Carcharias taurus* caught in Jeffreys Bay and compared the data with of “*L. natalensis*” in GenBank (FJ447375; FJ447440) (see Fig. 9). Calculated uncorrected pairwise distance between these two species is 0.002 (0.2%). On the basis of both molecular and morphological evidence, we consider that *L. natalensis* is a junior subjective synonym of *C. furcisetifer*. This synonymy extends the known geographical range of this parasite to include the eastern coast of South Africa.

This report further extends the range of chondrichthyan hosts used by *C. furcisetifer* to include *Carcharias taurus* and *Carcharinus leucas.* This is the first record for this species in southern Africa and further extends its geographical range to include the entire Indian Ocean basin.

***Caligus lalandei*** **Barnard, 1948**

*Syn. Caligus tenuicaudatus* Shiino, 1959

*Host*: *Seriola lalandi* Valenciennes, 1833

*Locality*: Struisbaai, South Africa (34°46′41.26″S, 20° 5′11.70″E), collected on 25 February 2010

*Material examined*: 39 females and 16 males. Vouchers: 26 females and 11 males deposited in the Iziko South African Museum, (SAMC-A088683); 13 females and 5 males in the Natural History Museum, London (NHMUK 2014.668-677).

*Representative DNA sequences*: GenBank: MW911365, MW925123

*Description*: Both sexes were redescribed by Ho et al. (2001).

*Remarks*: This is a very distinctive species characterized by the extreme development of the caudal rami in both sexes: in females the caudal rami are about 4.5 times longer than wide while in the male they are over 30 times longer (Ho et al., 2001). *Caligus lalandei* is host specific to the genus *Seriola* Cuvier, and has been reported from *S. hippos* Günther and *S. quinqueradiata* Temminck & Schlegel, as well as *S. lalandi*. Originally described from South Africa (Barnard, 1948), its known distribution outside of South African waters now includes Mexico (Shiino, 1959a), Chile (Baeza & Castro, 1982), New Zealand (Jones, 1988), Korea and Japan (Ho et al., 2001), and Australia (Hutson et al., 2007).

***Caligus lineatus*** **n. sp.**

*Type Host*: *Pomatomus saltatrix* (Linnaeus, 1766)

*Type Locality*: Table Bay (33°52′59.55″S, 18°25′41.51″E), collected on 05 May 2005

*Type Material*: Holotype female and 3 male paratypes deposited in the collections of the Iziko South African Museum (SAMC-A088684), and 1 female and 1 male paratype in the Natural History Museum (London) (NHMUK 2016.514-515).

*ZooBank number*: urn:lsid:zoobank.org:act: B6D877DD-7969-4956-B338-8143E4E0B468

*Etymology:* The species name *lineatus* alludes to the distinctive parallel lateral margins of the dorsal cephalothoracic shield of both sexes, and of the genital complex of the female.

**Figure 1:**
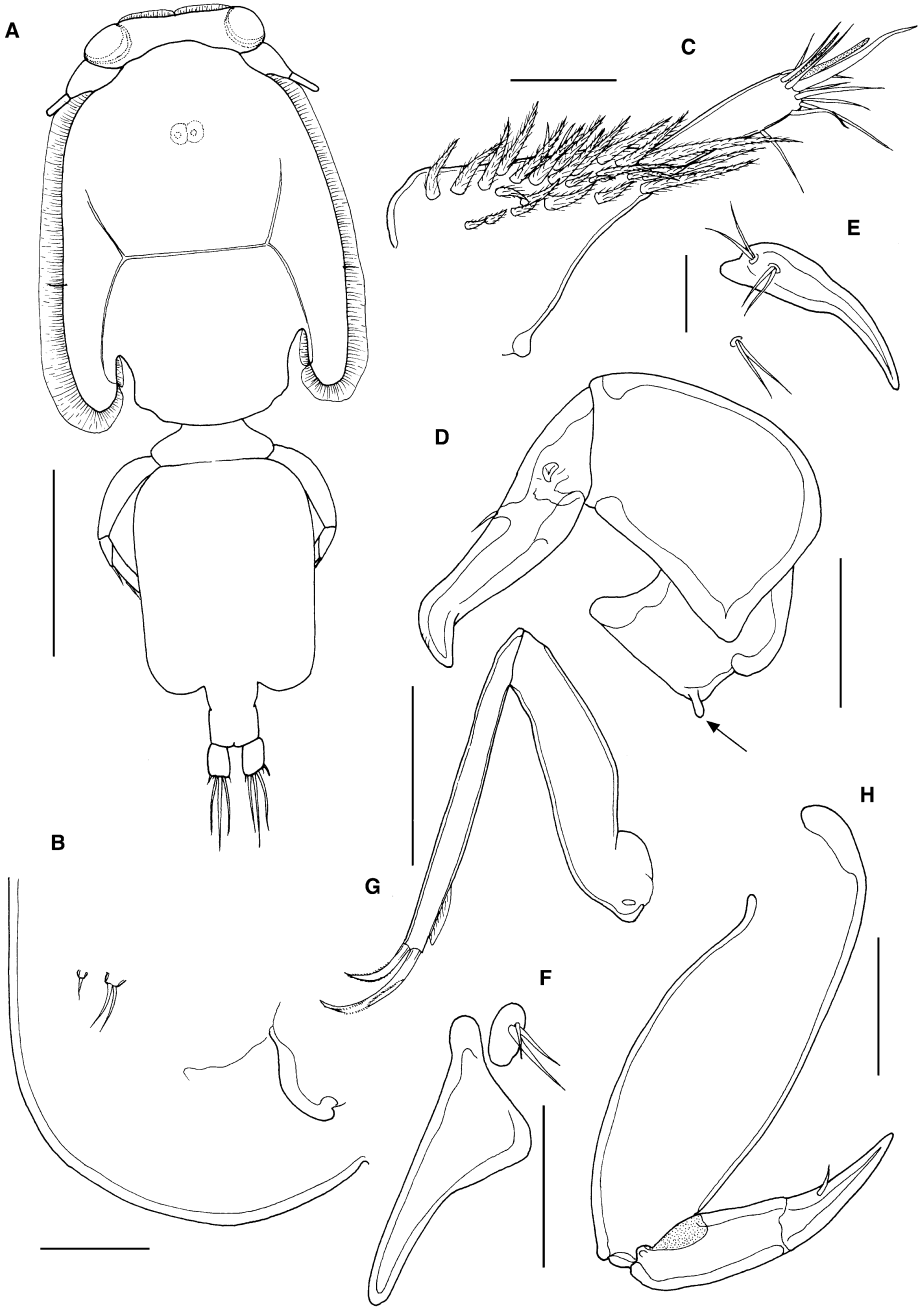
*Caligus lineatus* **n. sp.** female. A, habitus, dorsal (ornamentation of caudal setae omitted); B, posterolateral corner of genital complex, ventral view showing leg 5 and genital aperture; C, antennule, ventral; D, antenna, ventral; E, postantennary process, ventral; F, maxillule, G, maxilla; H, maxilliped. Scale bars: A, 1 mm, B-D, F-H, 100 μm, E, 50 μm.

*Description*: Adult females (Fig. 1A) body length 4.01 and 4.18 mm, including caudal rami. Cephalothorax elongate with marked posterior sinuses; about 1.45 times longer than wide (2.26 x 1.54 mm) and comprising about 54% of total body length. Free margin of thoracic portion of dorsal cephalothoracic shield extending posteriorly beyond rear margins of lateral portions. Genital complex 1.27 times longer than wide (1.21 x 0.95 mm); with straight, parallel lateral margins and rounded posterolateral angles (Fig. 1A). Copulatory pores paired, located on ventral surface of genital complex medial to fifth legs (Fig. 1B) and close to anterior corner of abdomen. Genital complex about 3.3 times longer than abdomen. Abdomen indistinctly 2-segmented; first somite wider than long (0.28 x 0.14 mm), second wider than long (0.21 x 0.14 mm); carrying paired caudal rami distally; anal slit terminal. Caudal rami with parallel sides, just wider than long, measured at midpoints of margins. Each ramus armed with short hirsute seta at inner distal angle, slightly longer hirsute seta at outer distal angle, minute hirsute seta located just ventral to outer distal seta, and 3 setae on distal margin (2 long and plumose; middle seta reduced, non-plumose).

Antennule (Fig. 1C) 2-segmented; large proximal segment with 25 plumose setae along anteroventral margin and 2 setae located dorsally; distal segment bearing 12 elements (11 setae plus 1 aesthetasc) around apex, plus isolated seta on posterior margin. Antenna (Fig. 1D) comprising proximal segment with minute, posteriorly-directed spinous process (arrowed in Fig. 1D); middle segment subrectangular, tapering slightly distally, unarmed; terminal segment forming short, weakly recurved claw bearing short spinous process proximally, and armed with slender distal seta on anterior margin. Post-antennal process (Fig. 1E) well-developed, slightly curved; ornamented with 2 bi-sensillate papillae on basal part and single similar bi-sensillate papilla on adjacent ventral cephalothoracic surface.

Mandible of typical stylet-like structure, with 12 marginal teeth. Maxillule (Fig. 1F) comprising anterior papilla bearing 3 unequal, naked setae and simple, posterior, tine-like process. Maxilla 2-segmented (Fig. 1G), comprising elongate syncoxa and basis: syncoxa unarmed; basis bearing subapical flabellum on anterior margin, and terminating in 2 unequal claw-like elements (calamus and canna): calamus longer than canna, both ornamented with strips of serrated membrane arranged obliquely around surface. Maxilliped subchelate (Fig. 1H); large proximal segment unarmed and lacking process on smooth myxal surface; distal subchela with apical claw separated from proximal segmental part by incomplete suture; claw armed with 1 small seta.

**Figure 2:**
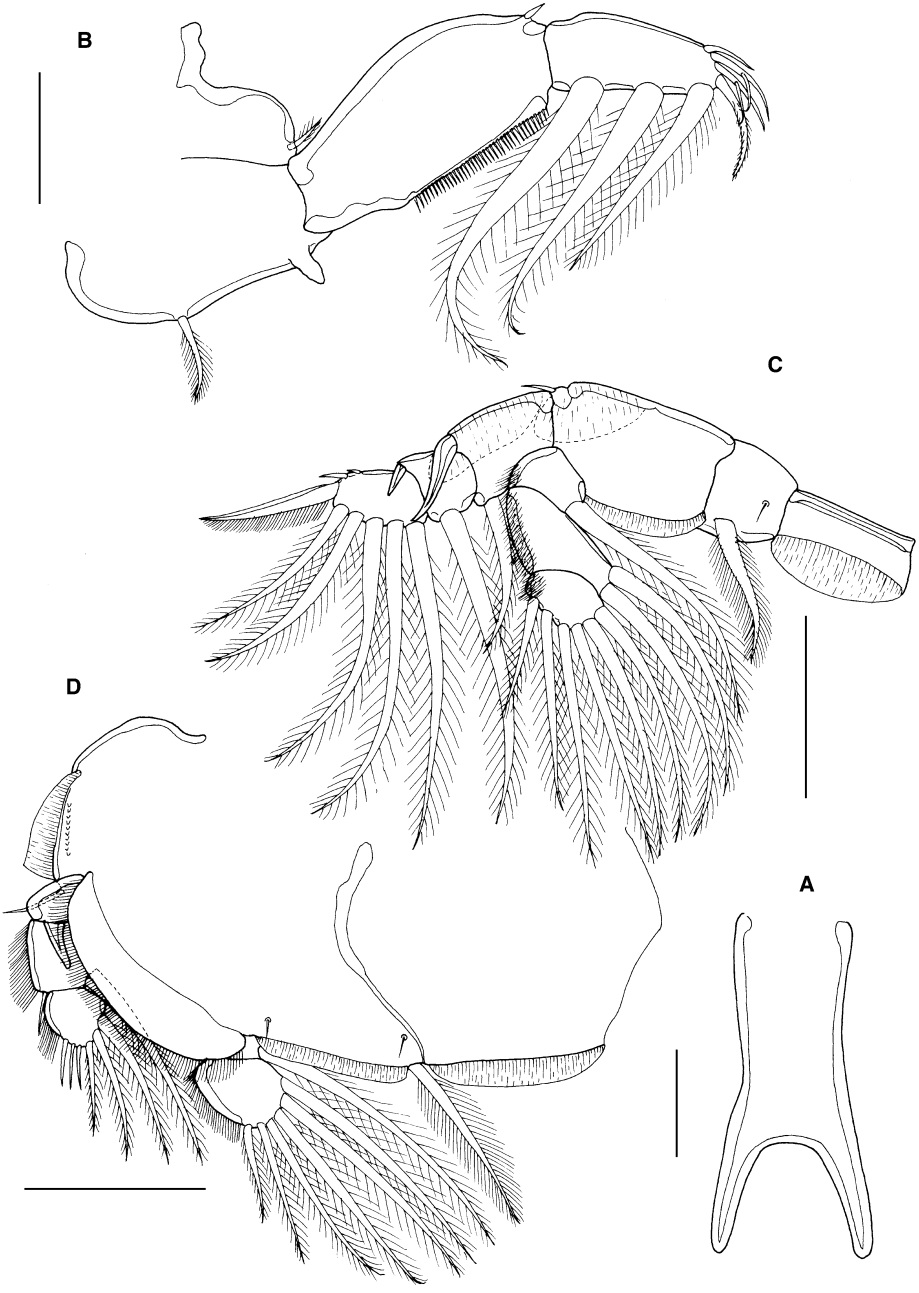
*Caligus lineatus* **n. sp.** female. A, sternal furca; B, leg 1, anterior; C, leg 2, ventral; D, leg 3, ventral. Scale bars: A-B, 100 μm, C-D, 250 μm.

Sternal furca (Fig. 2A) with long box bearing slightly divergent tines, each with bluntly rounded tip.

First swimming leg pair (Fig. 2B) with coxae joined by slender intercoxal sclerite (interpodal bar); basis with inner and outer plumose setae; exopod 2-segmented; endopod represented by unarmed process on posterior margin of basis. Exopod directed laterally and forming main axis of leg; first segment robust, about 2.1 times longer than wide and armed with small outer (anterior) spine and ornamented with setule row along posterior margin; second segment armed with 3 long plumose setae along posterior margin and 4 distal elements. Distal elements as follows: spine 1 (anterior-most) simple, just more than half as long as spines 2 and 3; latter each with accessory process; seta 4 about 25% longer than spines 2 and 3, and shorter than segment.

Second leg (Fig. 2C) biramous, with flattened protopodal segments and 3-segmented rami. Coxae of leg pair joined by narrow, plate-like, intercoxal sclerite bearing marginal membrane posteriorly. Coxa with plumose seta posteriorly plus surface sensilla. Basis armed with outer naked seta; ornamented with marginal membrane posteriorly, and flap of membrane anteriorly, reflexed back over dorsal surface of segment. Exopodal segments 1 and 2 each with large reflexed outer spines extending obliquely across ventral surface of ramus; segment 3 with 2 outer spines (proximal spine small), apical spine with marginal membrane laterally and pinnules medially, and 5 inner plumose setae. Endopodal segments 1 and 2 armed with 1 and 2 inner plumose setae respectively; segment 3 with 6 plumose setae; outer margins of all endopodal segments ornamented with fine setules.

Third leg pair (Fig. 2D) forming flattened plate closing posterior margin of cephalothoracic sucker, as typical for genus. Leg pair joined by plate-like intercoxal sclerite (apron) ornamented with marginal membrane posteriorly. Protopodal part flattened, bearing inner plumose seta posteriorly at junction with intercoxal plate, and outer plumose seta near base of exopod; sensillae located adjacent to inner coxal seta and adjacent to origin of endopod; ornamented with row of spinules near lateral margin, strip of membrane along posterior margin medial to endopod and along lateral margin anterior to exopod; space between rami covered by flap-like velum ornamented with row of fine setules along free margin. Exopod 3-segmented; first segment armed with short, weakly-curved outer claw directed over ventral surface of ramus; second segment with slender outer spine and inner plumose seta; third with 3 outer spiniform elements and 4 inner plumose setae (Innermost seta broken off in figured specimen); outer margins of segments 2 and 3 ornamented with rows of slender setules. Endopod 2-segmented; first segment with inner plumose seta; second with 6 setal elements increasing in length from outermost to innermost.

**Figure 3:**
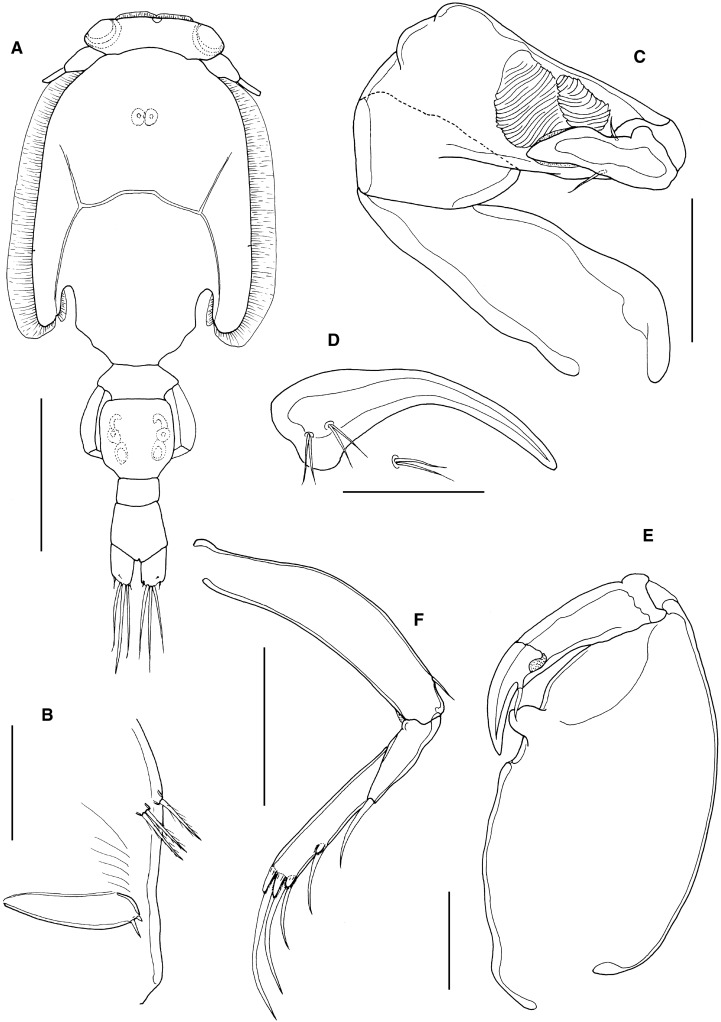
*Caligus lineatus* **n. sp.** male. A, habitus, dorsal (ornamentation of caudal setae omitted); B, posterolateral corner of genital complex, ventral view showing leg 5 and genital operculum representing leg 6; C, antenna; D, postantennary process, ventral; E, maxilliped; F, leg 4. Scale bars: A, 1 mm, B-F, 100 μm.

Fourth leg (as in male, see Fig. 3F) 3-segmented, comprising long protopodal segment and 2-segmented exopod with exopodal segments separated by oblique articulation: protopodal segment armed with outer seta; proximal exopodal segment with slender outer spine; compound distal segment armed with 1 lateral spine with pecten at base, plus 3 unequal naked spines along distal margin, each with pecten at base.

Fifth leg located posterolaterally on genital complex, represented by plumose, outer protopodal seta originating on papilla on somite surface and 2 plumose setae on small inner papilla representing exopod (Fig. 1B). Sixth leg represented by plate closing off genital opening.

Adult male (Fig. 3A) mean body length 3.69 mm (range 3.42 to 3.87 mm), including caudal rami (based on 3 specimens). Cephalothorax as in female. Genital complex wider than long (0.54 x 0.48 mm), measured along the mid-line; with more or less parallel lateral margins. Abdomen 2-segmented; first segment much shorter than wide (0.17 mm x 0.28 mm), second segment about twice as long as first, and about as long as wide (0.36 x 0.36 mm); carrying paired caudal rami distally; anal slit terminal. Caudal rami with parallel sides, just wider than long, measured at midpoints of margins. Each ramus armed with short hirsute seta at inner distal angle, slightly longer hirsute seta at outer distal angle, minute hirsute seta located just ventral to outer distal seta, and 3 setae on distal margin (2 long and plumose; middle seta reduced, non-plumose).

Antennules, mandible, maxillule and maxilla as in female. Antenna modified (Fig. 3C); first segment elongate; second segment reflexed, elongate, bearing corrugated adhesion pads ventrally in distal part; distal segment forming short powerful claw, armed with 2 setae proximally. Post-antennal process (Fig. 3D) similar to female but more curved; ornamented with bi-sensillate papillae as in female.

Maxilliped (Fig. 3E) with rounded myxal process on robust proximal segment and with single pore on surface proximal to myxal process and directly opposing tip of claw.

Leg 1 to leg 4 (Fig. 3F) as in female.

Leg 5 (Fig. 3B) represented by plumose, outer protopodal seta originating on papilla on somite surface and 2 plumose setae on inner papilla representing exopod. Sixth leg represented by plate closing off genital opening; armed with 1 seta and 1 short spine on outer distal corner of genital operculum.

*Remarks*: *Caligus lineatus* **n. sp.** has a 3-segmented leg 4 with 4 spines on the compound distal exopodal segment (Fig. 3F). It shares this fourth leg type with about 90 other species of *Caligus*. Although the shape of the genital complex of the adult female can vary with reproductive state, the length:width ratios and proportional lengths of the genital complex and abdomen of the new species are distinctive: the genital complex and the abdomen of the new species are both longer than wide, and the genital complex is more than 3 times longer than the abdomen. Only four other species of *Caligus* share this configuration: *C. asymmetricus* Kabata, 1965, *C. ocyurus* Cressey, 1991, *C. xystercus* Cressey, 1991 and *C. zei* Norman & Scott, 1906. The new species and *C. asymmetricus* both share an unusual feature, the possession of a tiny posterior process (arrowed on Fig. 1D) on the first segment of the antenna in the female. However, they differ in numerous features, for example: the new species has widely spaced and divergent tines on the sternal furca, whereas the furca is tiny (almost vestigial) and has almost parallel tines originating very close together in *C. asymmetricus*; the outer margin of the second endopodal segment of leg 2 is ornamented with setules in the new species but with large denticles in *C. asymmetricus*; and the maxilliped of the female has a smooth myxal margin in the new species but carries a distinct process in *C. asymmetricus* (Cressey & Cressey, 1980).

The new species differs from *Caligus zei* as redescribed by Kabata (1979) in numerous features: the spines on leg 4 are much longer in the new species than in *C. zei*, the maxilliped of the female has a smooth myxal margin in the new species but carries a distinct process in *C. zei* and the proportional lengths of the setal elements on the distal margin of the exopod of leg 1 are different. The apical claw of the antenna of the male of the new species is simple but in *C. zei* it consists of two spatulate blades.

The females of both *C. ocyurus* and *C. xystercus* are somewhat similar in general shape to the new species: all three have a subrectangular dorsal cephalothoracic shield and a genital complex with parallel rather than rounded convex lateral margins. These three species appear closely related and share numerous fine scale characteristics. *Caligus ocyurus* shares a particularly close resemblance in gross body form (cf. Fig. 1A and Cressey, 1991: Fig 127). However, the new species has a tiny posterior process on the first segment of the female antenna compared to a large spinous process present in both *C. ocyurus* and *C. xystercus.* There are additional differences in shape of the postantennal process, maxillule, and sternal furca that serve to separate the new species from *C. ocyurus*, and the spines on leg 4 are markedly longer in the new species than in *C. ocyurus*. *Caligus xystercus* differs slightly from the other two species in body shape, because its dorsal cephalothoracic shield is slightly wider posteriorly and the genital complex is only 1.25 times longer than wide (compared to 1.40 to 1.45 times). However, the relative lengths of the spines on leg 4 are very similar in the new species and *C. xystercus*, and the shapes of the postantennal process, maxillule, and sternal furca are the same. The females can best be distinguished by the process on the antenna and by the proportions of the genital complex. The male of *C. xystercus* is unknown.

*Caligus ocyurus* was first reported from a lutjanid, *Ocyurus chrysurus* Bloch, caught off Belize (Cressey, 1991). *Caligus xystercus* was also first reported from Belize, from a remarkably wide range of aulostomid, haemulonid, lutjanid, pomacanthid, priacanthid and sparid host fishes (Cressey, 1991). Neither of these two species has previously been reported from *Pomatomus saltatrix*.

Two species recently described from Japanese waters, *C. chinlonglini* Ohtsuka & Boxshall, 2019 and *C. kajii* Ohtsuka & Boxshall, 2019, share some features with *C. lineatus* **n. sp.** but can be distinguished by the form of the fifth and sixth legs in the adult male. Both Japanese species have these legs defined as processes visible on the posterolateral margins of the genital complex, whereas the male of the new species lacks such processes. In addition the genital complex of the female of *C. kajii* is subquadrate (1.14 times longer than wide) and the abdomen is 1-segmented, compared to elongate (1.27 times longer than wide) and 2-segmented, respectively, in *C. lineatus* **n. sp.** The female is unknown in *C. chinlonglini* but the male carries 2 processes on the myxal margin of the maxilliped compared to a single process in *C. lineatus* **n. sp.**

In southern African waters *P. saltatrix* has been listed as the host for three species of *Caligus*: *C.* cf. *affinis* Heller, 1866 (Kensley & Grindley, 1973), *C. coryphaenae* Steenstrup & Lütken, 1861 (Oldewage, 1992; Oldewage & Avenant-Oldewage, 1993), and *C. mauritanicus* Brian, 1924 (Barnard, 1955). These records were all included in the checklist of Dippenaar (2005). In 1955 Barnard (1955: 310) listed the name of *C. mauritanicus* in brackets indicating that the record was not from South Africa, but was from elsewhere on the African continent, presumably based on Brian’s original report of *C. mauritanicus* from Mauritania (Brian, 1924). Oldewage & Van As (1989) erroneously attributed this record to Barnard (1955) as an original report from False Bay, South Africa. Özak et al. (2010) re-examined Brian’s material of *C. mauritanicus* from *Pomatomus saltatrix*, reported as var. *temnodontis* by Brian (1924), and considered that this named variety represents a valid species, *C. temnodontis* Brian, 1924, known only from *Pomatomus saltatrix*. They also referred the *Caligus* cf. *affinis* reported by Kensley & Grindley (1973) to *C. temnodontis.*

At present only two *Caligus* species are known from *P. saltatrix* in South Africa: *C. coryphaenae* and *C. temnodontis. Caligus lineatus* **n. sp.** is readily distinguishable from the former by its short, indistinctly 2-segmented abdomen compared to the large, apparently 3-segmented abdomen of *C. coryphaenae* (Ho & Lin, 2004). In addition, *C. coryphaenae* is characterized by the presence of accessory processes either side of the sternal furca which are lacking in the new species. The new species possesses 3 plumose setae on the posterior margin of the distal exopod segment of leg 1 whereas *C. temnodontis* belongs to the *C. productus* group, characterized by the loss of these setae (Boxshall & El-Rashidy, 2009).

***Caligus longipedis*** **Bassett-Smith, 1898**

*Syn. Caligus amplifurcus* Pearse, 1953

*Caligus lucidus* Heegaard, 1962

*Caligus rugosus* Shiino, 1959

*Host*: *Pseudocaranx dentex* (Bloch & Schneider, 1801)

*Locality*: Ushaka Sea World, South Africa (29°52′4.53″S, 31° 2′44.30″E), collected on 18 March 2004

*Material examined*: 36 females and 4 males. Vouchers: 27 females and 2 males in the Iziko South African Museum, (SAMC-A088685); 9 females and 2 males in the Natural History Museum, London (NHMUK 2015.488-497).

*Description*: A modern detailed description of the female is available in Ho & Lin (2004) and for the male, in Venmathi Maran et al. (2009).

*Remarks*: *Caligus longipedis* was originally described by Bassett-Smith (1898) based on material taken from *Caranx melampygus* Cuvier collected off Aden. It was later redescribed from the same host taken off Hawaii by Lewis (1967), who also recognized both *C. amplifurcus* Pearse, 1953 (from *Caranx crysos* (Mitchill) in Florida, USA) and *C. lucidus* Heegaard, 1962 (from *Nelusetta ayraud (Quoy & Gaimard)* (as *Cantherines ayraudi*) in Australian waters off New South Wales) as junior subjective synonyms. The record of Ho & Lin (2004) is based on material collected in Taiwan from *Megalaspis cordyla* Linnaeus. However the male of *C. longipedis* described by Ho & Lin (2004) is not conspecific with the female. The male of *C. longipedis* was described by Venmathi Maran et al. (2009). Ho & Lin (2004) provided a full list of published records of *C. longipedis* from numerous additional hosts, including carangids and several other families. Boxshall (2018) reported *C. longipedis* from juvenile *Gerres* spp. in Moreton Bay, Australia. It has previously been reported from *Pseudocaranx dentex* in Japanese waters as *Caligus rugosus* Shiino, 1959 (Shiino, 1959b) and as *C. amplifurcus* (Shiino, 1959b; Kubota & Takakuma, 1963).

This widespread species is known from Yemen, Australia, Belize, USA (Florida, Hawaii), Mexico (Pacific coast), India, Japan, Taiwan and the eastern Pacific. This is the first record from South Africa.

***Caligus rufimaculatus*** **Wilson, 1905**

*Host*: *Lagocephalus sceleratus* (Forster, 1788); *Zanclus cornutus* (Linnaeus, 1758)

*Locality*: Ushaka Sea World, South Africa (29°52′4.53″S, 31° 2′44.30″E), collected on 9 September 2004 from *L sceleratus*; collected on 28 August 2004 from *Zanclus cornutus*

*Material examined*: 6 females and 1 male. Vouchers: 4 females from *Z. cornutus* deposited in the Iziko South African Museum, (SAMC-A088686); 2 females and 1 male from *Z. cornutus* in the Natural History Museum, London (NHMUK 2014.756-758).

*Description*: This species was redescribed by Cressey (1991) based on re-examination of the type material.

*Remarks*: The distribution of this species is centred on the Atlantic and Gulf coasts of the eastern USA, where it has been reported from a range of coastal elasmobranch and actinopterygian fishes including: *Fundulus majalis* (Walbaum), *F. heteroclitus* (Linnaeus), *Mugil cephalus* Linnaeus (Wilson 1905, 1908), *Pomatomus saltatrix*, *Mugil* sp., *Oligoplites saurus* (Bloch & Schneider), *Mobula hypostoma* (Bancroft), *Aetobatus narinari* (Euphrasen) (as *Stoasodon narinari*), *Chaetodipterus faber* (Broussonet), *Strongylura* sp., *Pseudobatos lentiginosus* (Garman) (as *Rhinobatos lentiginosus* Garman) and *Eucinostomus* *jonesii* (Günther) (as *Eucinostomus pseudogula* Poey) (Bere, 1936). Bere (1936) also reported it free-swimming in the plankton. Cressey (1991) added several additional hosts: *Eucinostomus gula* (Quoy & Gaimard), *Centropristis striata* (Linnaeus) (as *C. melana* Ginsberg), *Diplodus holbrookii* (Bean), *Haemulon plumierii* (Lacepède), *Acanthostracion quadricornis* (Linnaeus) (as *Lactophrys quadricornis*), *Lagodon rhomboides* (Linnaeus), *Lutjanus synagris* (Linnaeus), *Stephanolepis hispida* (Linnaeus) (as *Monacanthus hispidus* Linnaeus), *Nicholsina usta* (Valenciennes) and *Orthopristis chrysoptera* (Linnaeus). Cressey (1991) confirmed that this species was confined to the Atlantic coast of the USA and the southwest coast of Florida. This is the first record for this species in southern Africa.

***Caligus tenuis*** **(van Beneden, 1852)**

*Syn. Sciaenophilus tenuis* van Beneden, 1852

*Host*: *Argyrosomus japonicus* (Temminck & Schlegel, 1843)

*Locality*: Witsand (34°24′8.19″S, 20°48′59.50″E), collected on 15 November 2002

*Material examined*: 2 females – both used, unsuccessfully, for molecular study.

*Description*: A detailed redescription of the female of *Caligus tenuis* (as *Sciaenophilus tenuis*) was provided by Dojiri & Ho (2013).

*Remarks*: This widely distributed parasite was originally described from European waters (van Beneden, 1852) but has since been reported from both sides of the Atlantic Ocean including the Gulf of Mexico in the west and the Mediterranean in the east, and from India and Sri Lanka in the Indian Ocean (geographical records summarised in Dojiri & Ho, 2013, as *Sciaenophilus tenuis*).

The host records of *C. tenuis* were also summarised by Dojiri & Ho (2013). This parasite predominantly uses sciaenid hosts and has been reported from at least ten species, including: *Argyrosomus regius* (Asso), *A. hololepidotus* (Lacepède), *Larimus fasciatus* Holbrook, *Nibea maculata* (Bloch & Schneider), *Otolithoides biauritus* (Cantor), *Pogonias cromis* (Linnaeus), *Sciaena umbra* Linnaeus, *Protonibea diacanthus* (Lacepède), and *Umbrina cirrosa* (Linnaeus), as well as from *Johnius* sp. In addition, it has been reported from the non-sciaenid *Lobotes surinamensis* (Bloch) (Cressey & Nutter, 1987) although it seems highly likely that this is a misidentification of *Caligus macrurus* Heller, 1865, a widespread parasite of this host (Özak et al., 2017). *Argyrosomus japonicus* (Temminck & Schlegel) is a known host for *C. tenuis* (as *Sciaenophilus tenuis*) in South African waters (Grobler et al., 2003).

***Caligus tetrodontis*** **Barnard, 1948**

*Host*: *Amblyrhynchotes honckenii* (Bloch, 1785)

*Localities*: Struisbaai (34°46′41.26″S, 20° 5′11.70″E), collected on 28 December 2006 and 03 April 2007

Tsitsikamma National Park (34°01′13″S, 23°52′35″E), collected on 10 March 2013

*Material examined*: 5 females and 3 males. Vouchers: 4 females and 2 males from *Amblyrhynchotes honckenii* deposited in the Iziko South African Museum, (SAMC-A08867); 1 female and 1 male from *Amblyrhynchotes honckenii* in the Natural History Museum, London (NHMUK 2014.678-679).

*Representative DNA sequences*: GenBank: MW911366, MW925124 (specimen sequenced was from *A. honckenii* caught in Tsitsikamma National Park).

*Supplementary description*: Mean body length of females from *Amblyrhynchotes honckenii* examined here: 5.08 mm (range 4.53 to 5.31 mm (based on four specimens). The sample contained another ovigerous female with a body length of 4.31 mm, but it had a shrivelled genital complex and was not included in the body length calculations.

**Figure 4:**
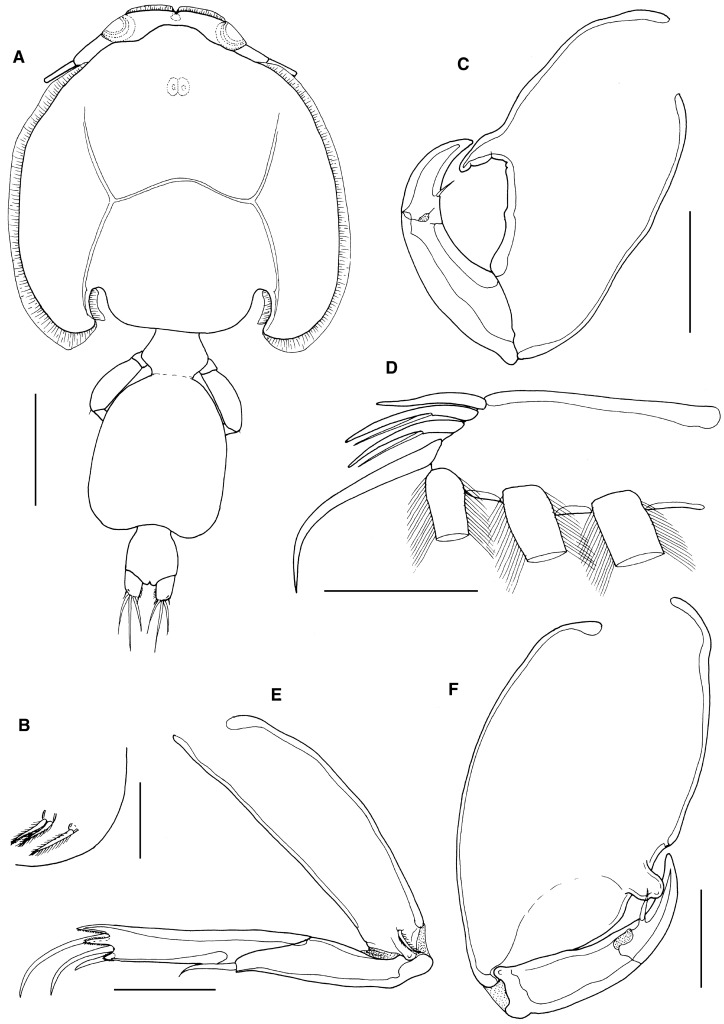
*Caligus tetrodontis* Barnard, 1948. A, female habitus, dorsal (ornamentation of caudal setae omitted); B, posterolateral corner of genital complex, ventral view showing leg 5; C, female maxilliped; D, tip of exopod of leg 1; E, leg 4; F, male maxilliped. Scale bars: A, 1 mm, B, 200 μm, C, E, 250 μm, D, F 100 μm.

Adult female (Fig. 4A) dorsal cephalothoracic shield subcircular (length 2.90 mm, width 2.92 mm): genital complex with rounded posterolateral corners, about 1.12 times longer than wide; length along mid-line 1.36 mm, maximum width 1.21 mm. Genital complex length 1.37 mm, width 1.20 mm, about 1.14 times longer than wide; with more-or-less parallel lateral margins and evenly rounded posterolateral corners. Abdomen 1-segmented, length 0.57 mm, maximum width 0.47 mm, about 1.21 times longer than wide. Fifth legs not visible in dorsal view; comprising outer (protopodal) papilla bearing single plumose seta and inner exopodal papilla bearing 2 plumose setae (Fig. 4B).

Antennule typical for genus. Antenna bearing short posterior process on proximal segment; distal claw strongly recurved. Postantennal process strongly curved, associated papillae each bearing single sensilla. Mandible typical for genus. Maxillule with simple posterior process. Maxilla typical for genus. Maxilliped (Fig. 4C) with short, strongly curved claw opposing elongate process on myxal surface of corpus.

Leg 1 typical for genus, with 3 plumose setae on posterior margin of distal exopodal segment; distal margin of segment (Fig. 4D) armature comprising long spine 1 lacking accessory process; spines 2 and 3 just longer than spine 1, each with long accessory process; seta 4 about twice as long as longest spine but just shorter than segment. Leg 2 with outer margin of second endopodal segment ornamented with slender setules; outer spines on first and second exopodal segments reflexed obliquely across surface of ramus. Leg 3 without ornamentation on surface of apron; outer spine on first exopodal segment slightly curved, not reaching level of articulation with second exopodal segment. Leg 4 (Fig. 4E) 3-segmented: coxobasis bearing single outer distal seta; proximal exopodal segment with naked outer spine; distal exopodal segment with 3 distal spines, decreasing in length from inner to outer: each of distal spines with elongate pecten rigidly fused to segment at base.

Mean body length of males from *Amblyrhynchotes honckenii* examined here 4.51 and 4.53 mm (based on 2 specimens). Male maxilliped (Fig. 4F) with myxal process slightly shorter and broader than in female.

*Remarks*: *Caligus tetrodontis* was briefly described by Barnard (1948) based on material collected from *Torquigener hypselogeneion* (Bleeker) [as *Tetrodon hypselogeneion*] caught off Port Elizabeth (South Africa). Barnard (1948) provided only four figures: the posterior part of the body from the fifth pedigerous somite back (in both sexes), the sternal furca, and the tip of the fourth leg. He subsequently re-used the first three of these figures (Barnard, 1955), but gave no further morphological detail. If the original description (Barnard, 1948) was inadequate then the subsequent redescription by Oldewage (1990) is even less informative. Oldewage (1990) redescribed the female of a caligid identified as *C. tetrodontis* on the basis of material taken from *Arothron hispidus* Linnaeus, 1758 caught off the Transkei coast (South Africa). The line drawings provided by Oldewage (1990) lack useful detail and generate confusion: Oldewage’s paper is entitled “A redescription of female *Caligus tetrodontis*…” but his material clearly included adult males, given that the scanning electron micrograph in his Figure 2c shows the tip of a male antenna.

The identity of Oldewage’s (1990) material requires confirmation because of a significant difference in female body size: his material was 2.82 mm in total length, whereas the body length of the type material was given as 4 to 5 mm (Barnard, 1948). The mean body lengths of the material from *Amblyrhynchotes honckenii* examined here were 5.08 mm for the female and 4.52 mm for the male. Oldewage’s, Barnard’s and our material all comes from South Africa, so it seems very unlikely that the size variation could be geographically based.

The material studied here from *A. honckenii* has the same body size as *Caligus tetrodontis* of Barnard (1948) and the morphological details conform to those given in the basic description of Barnard (1948). The rigidly-fused pectens on the tip of leg 4 are particularly distinctive. The two confirmed hosts of this taxon are the type host *Torquigener hypselogeneion* and *A. honckenii* reported here and by Oldewage & Van As (1989). The taxon reported by Oldewage (1990) from *Arothron hispidus* differs in the much smaller female body size and in having a short bifid myxal process on the female maxilliped (Oldewage, 1990: Fig.1h) compared to simple but elongate myxal process on the female maxilliped (Fig. 4C) in our material. The Oldewage material should be re-examined and its identity confirmed as we consider that it may represent a different, possibly new, species.

The record of *C. tetrodontis* from Brazil (cf. Boxshall & Montú, 1997) was based on a single male *Caligus* found in the plankton off the southern coast of Brazil and identified by Montú (1982). The Brazilian male differs from the South African male from *A. honckenii* in the size and shape of the myxal process on the maxilliped, in the proportions of the two free abdominal somites, and in the form of the antenna. We conclude that this Brazilian male is incorrectly identified, it is not *C. tetrodontis*.

***Caligus tumulus*** **n. sp.**

*Type Host*: *Chrysoblephus cristiceps* (Valenciennes, 1830)

*Type Locality*: Struisbaai (34°46′41.26″S, 20°5′11.70″E), collected on 03 April 2006

*Type Material*: Holotype female deposited in the collections of the Iziko South African Museum (SAMC-A088688); allotype male in the Natural History Museum (London) (NHMUK 2014.755).

*ZooBank number*: urn:lsid:zoobank.org:act: 3BB68992-D86D-4322-B23A-DAB682A03493

*Etymology:* The species name comes from the Latin *tumulus*, meaning a hillock, and refers to the paired accessory processes located either side of the sternal furca on the ventral surface of the cephalothorax in both sexes.

**Figure 5:**
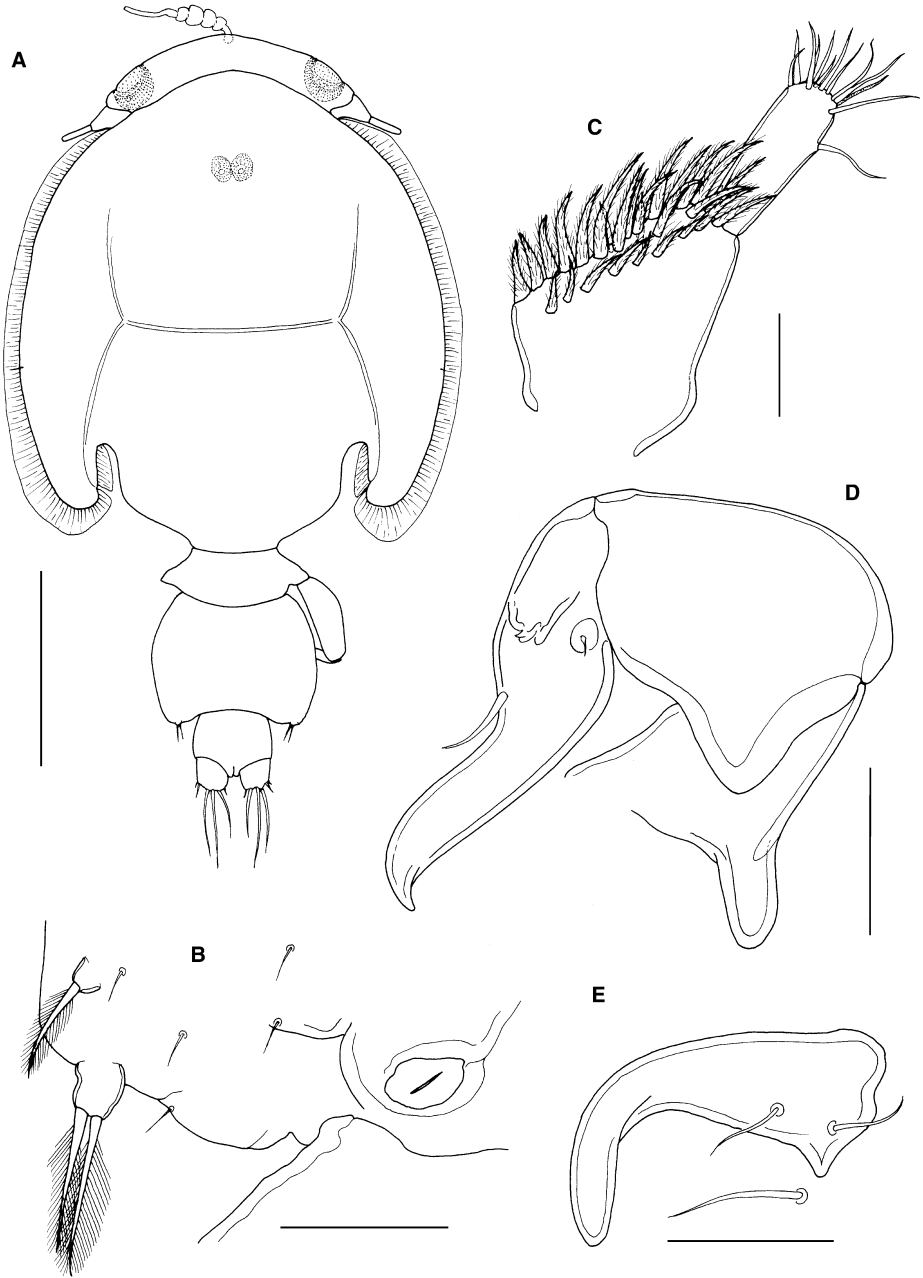
*Caligus tumulus* **n. sp.** female. A, habitus, dorsal (ornamentation of caudal setae omitted); B, posterolateral corner of genital complex, ventral view showing leg 5 and genital aperture; C, antennule, ventral; D, antenna, ventral; E, postantennary process, ventral. Scale bars: A, 1 mm, B-E, 100 μm.

*Description*: Holotype adult female (Fig. 5A) body length including caudal rami 3.91 mm, still attached via frontal filament indicating shape of genital complex possibly subject to change with reproductive status. Cephalothorax subcircular with marked posterior sinuses; just longer than wide (2.68 x 2.18 mm) and comprising about 69% of total body length. Free margin of thoracic portion of dorsal cephalothoracic shield extending posteriorly beyond rear margins of lateral portions. Genital complex wider than long (0.52 x 0.85 mm); with convex, rounded lateral margins and slight posterolateral lobes (Fig. 1A). Copulatory pores paired, located on ventral surface of genital complex medial to fifth legs and close to anterior corner of abdomen (Fig. 5B). Abdomen 1-segmented; wider than long (0.40 x 0.35 mm); carrying paired caudal rami distally; anal slit terminal. Caudal rami with parallel sides, just wider than long, measured at midpoints of margins. Each ramus armed with short hirsute seta at inner distal angle, slightly longer hirsute seta at outer distal angle, minute hirsute seta located just ventral to outer distal seta, and 3 setae on distal margin (2 long and plumose; middle seta reduced, non-plumose). Inner margin of ramus ornamented with setules as in male (cf. Fig. 8B).

Antennule (Fig. 5C) 2-segmented; large proximal segment with 25 plumose setae along anteroventral margin and 2 setae located dorsally; distal segment bearing 12 elements (11 setae plus 1 aesthetasc) around apex, plus isolated seta on posterior margin. Antenna (Fig. 5D) comprising proximal segment with posteriorly-directed spinous process; middle segment subrectangular, tapering slightly distally, unarmed; terminal segment forming strong, recurved claw bearing irregular spinous process and minute seta proximally, and armed with slender distal seta on anterior margin. Postantennal process (Fig. 5E) well-developed, recurved and claw-like; ornamented with 2 tiny unisensillate papillae on basal part and with single similar unisensillate papilla on adjacent ventral cephalic surface.

**Figure 6:**
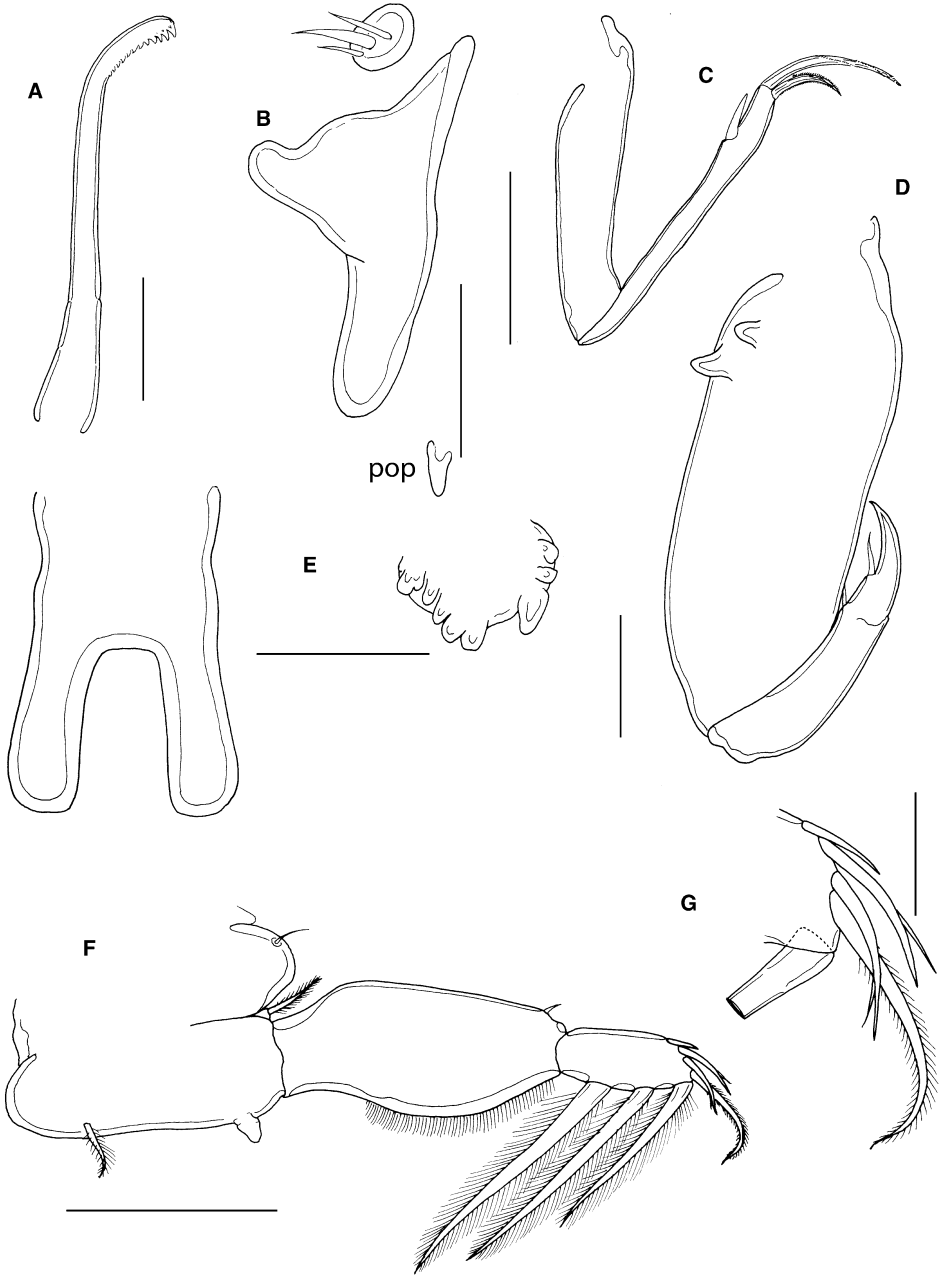
*Caligus tumulus* **n. sp.** female. A, mandible; B, maxillule and post-oral process (pop), C, maxilla; D, maxilliped; E, sternal furca and accessory process on left side, *in situ*; F, leg 1; G, tip of exopod of leg 1. Scale bars: A, 50 μm, B-E, 100 μm, F, 250 μm, G, 50 μm.

Mandible (Fig. 6A) of typical stylet-like structure, with 12 marginal teeth. Maxillule (Fig. 2B) comprising anterior papilla bearing 3 unequal, naked setae and simple, posterior, tine-like process. Small post-oral process present (Fig. 6B). Maxilla 2-segmented (Fig. 6C), comprising elongate syncoxa and basis: syncoxa unarmed; basis bearing subapical flabellum on anterior margin, and terminating in 2 unequal claw-like elements (calamus and canna). Calamus about twice as long as canna, both ornamented with strips of serrated membrane running obliquely around surface. Maxilliped subchelate (Fig. 6D); large proximal segment unarmed but with 2 proximal processes on posterior surface; distal subchela with apical claw separated from proximal segmental part by incomplete suture; segmental part and claw each armed with 1 seta.

Sternal furca (Fig. 6E) with long, slightly divergent tines, each with bluntly rounded tip; paired accessory processes located either side of furca, each with irregular lobulate surface.

First swimming leg pair (Fig. 6F) with unarmed coxae joined by slender intercoxal sclerite (interpodal bar); basis with inner and outer plumose setae; exopod 2-segmented; endopod represented by unarmed process on posterior margin of basis. Exopod directed laterally and forming main axis of leg; first segment robust, about 2.2 times longer than wide and armed with small outer (anterior) spine and ornamented with setule row along posterior margin; second segment armed with 3 long plumose setae along posterior margin and 4 distal elements (Fig. 6G). Distal elements as follows: spine 1 (anterior-most) small, simple, half as long as spines 2 and 3; latter each with accessory process; seta 4 about twice as long as spines 2 and 3, and about equal in length to segment.

**Figure 7:**
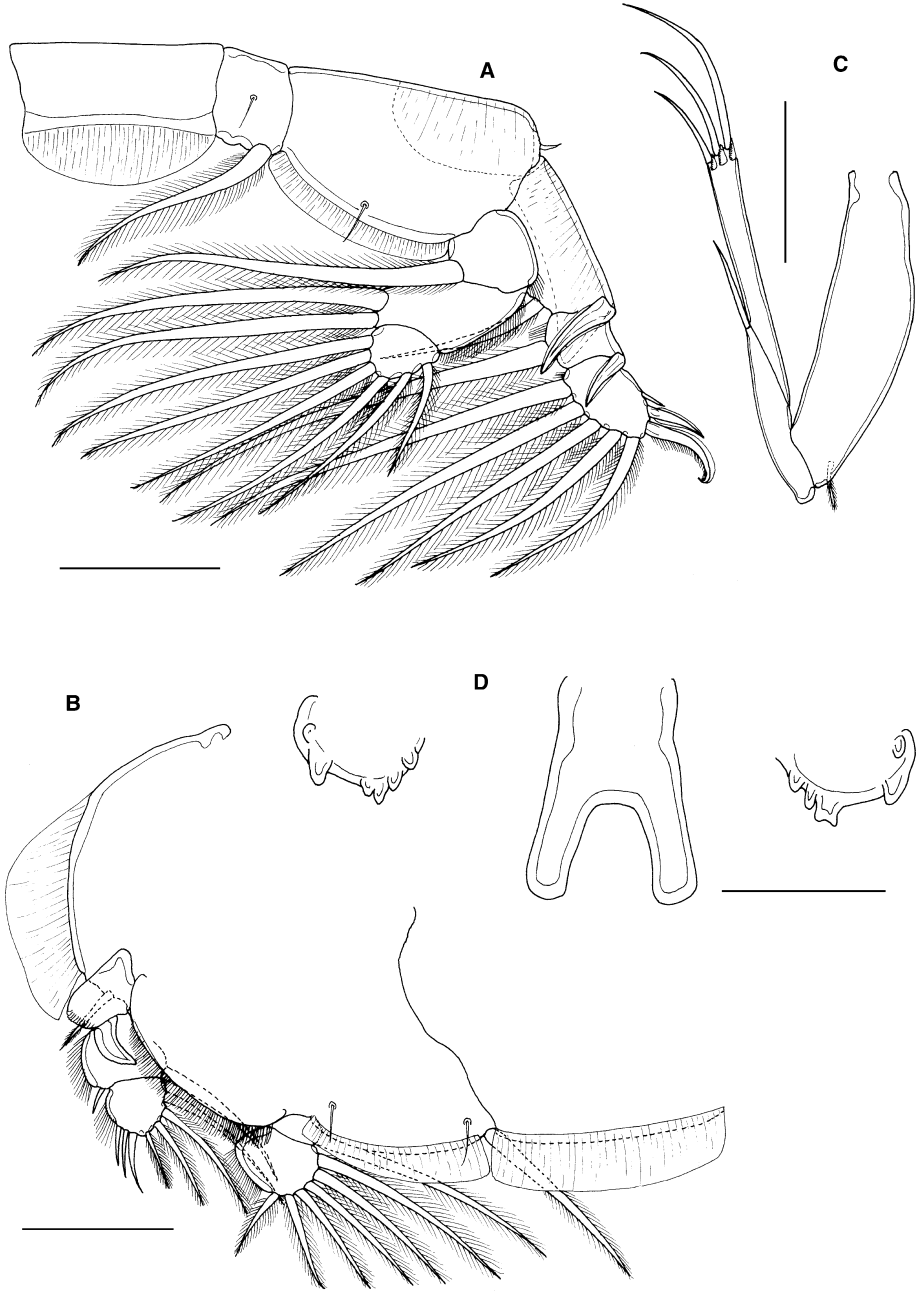
*Caligus tumulus* **n. sp.** female. A, leg 2; B, leg 3; C, leg 4. Male, D, sternal furca and accessory processes on both sides, *in situ*. Scale bars: A-B, D, 100 μm, C, 250 μm.

Second leg (Fig. 7A) biramous, with flattened protopodal segments and 3-segmented rami. Coxae of leg pair joined by narrow, plate-like, intercoxal sclerite bearing marginal membrane posteriorly. Coxa with plumose seta and surface sensilla. Basis armed with outer naked seta; ornamented with surface sensilla, marginal membrane posteriorly, and flap of membrane anteriorly, reflexed back over dorsal surface of segment. Exopodal segments 1 and 2 each with large reflexed outer spines extending obliquely across ventral surface of ramus; segment 3 with 2 outer spines (proximal spine small; distal spine with bilateral membrane), apical spine with marginal membrane laterally and pinnules medially, and 5 inner plumose setae. Endopodal segments 1 and 2 armed with 1 and 2 inner plumose setae respectively; segment 3 with 6 plumose setae; outer margins of first and second endopodal segments ornamented with fine setules.

Third leg pair (Fig. 7B) forming flattened plate closing posterior part of cephalothoracic sucker as typical for genus. Leg pair joined by plate-like intercoxal sclerite (apron) ornamented with marginal membrane posteriorly. Protopodal part flattened, bearing inner plumose seta at junction with intercoxal plate, and outer plumose seta near base of exopod; sensillae located adjacent to inner coxal seta and adjacent to origin of endopod; ornamented with membrane along posterior margin medial to endopod and along lateral margin anterior to exopod; space between rami covered by flap-like velum ornamented with row of fine setules along free margin. Exopod 3-segmented; first segment with rugose surface markings laterally, armed with weakly curved, outer claw directed over ventral surface of ramus; second segment with slender outer spine and inner plumose seta; third with 7 setal elements (3 outer spiniform elements and 4 inner plumose setae); outer margins of segments 2 and 3 ornamented with rows of slender setules. Endopod 2-segmented; first segment with inner plumose seta; second with 6 setal elements increasing in length from outermost to innermost.

Fourth leg (Fig. 7C) 3-segmented, comprising large protopodal segment and 2-segmented exopod with exopodal segments separated by oblique articulation: protopodal segment armed with outer seta; first exopodal segment with slender outer spine; second segment armed with 3 unequal naked spines along distal margin, each with pecten at base.

Fifth leg located posterolaterally on genital complex, represented by plumose, outer protopodal seta originating on papilla on somite surface and 2 plumose setae on small inner papilla representing exopod (Fig. 5B). Sixth leg represented by unarmed plate closing off genital opening.

**Figure 8:**
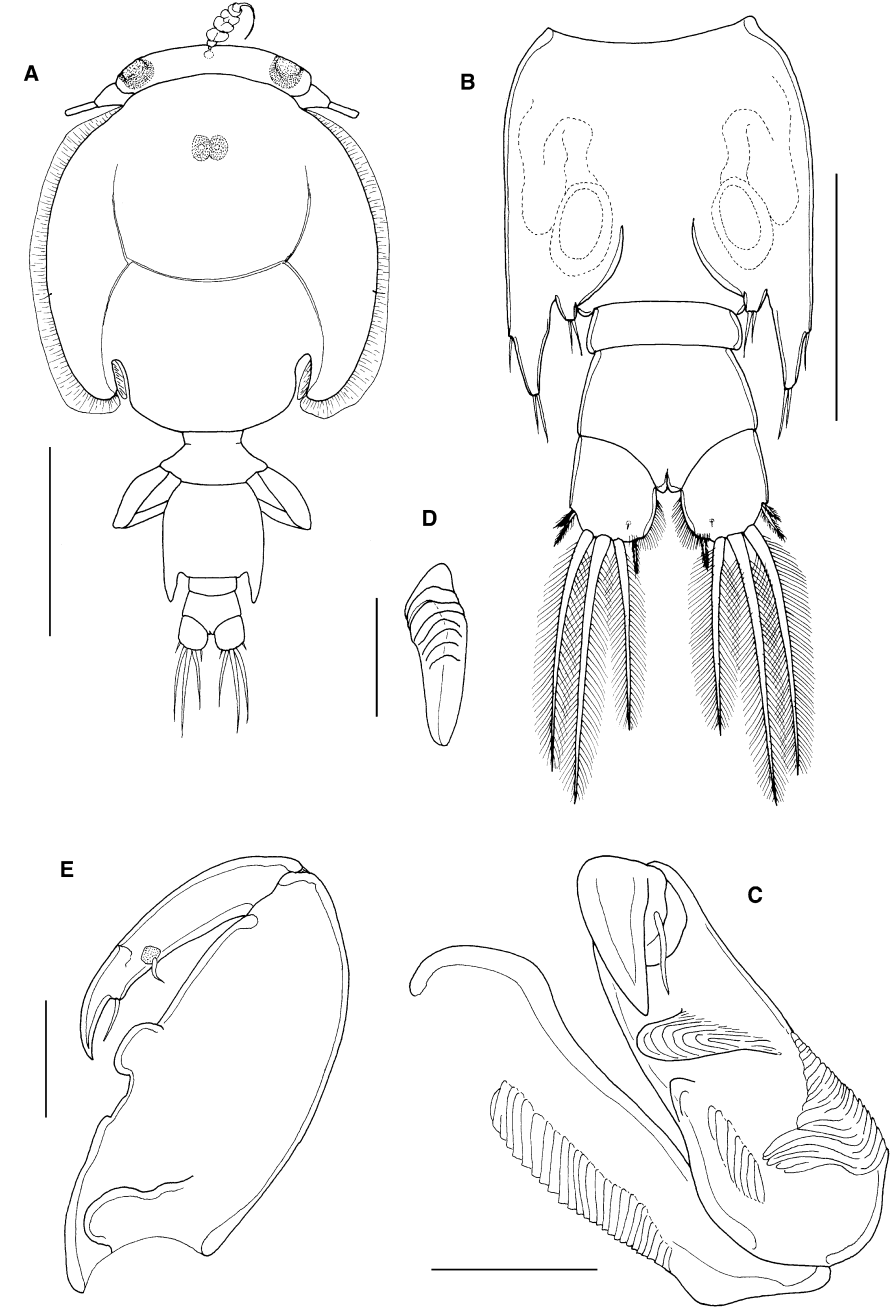
*Caligus tumulus* **n. sp.** male. A, habitus, dorsal (ornamentation of caudal setae omitted); B, genital complex, ventral; C, antenna; D, post-oral process, ventral; E, maxilliped. Scale bars: A, 1 mm, B 0.5 mm, C, E, 100 μm, D, 50 μm.

Allotype adult male (Fig. 8A) body length including caudal rami 3.20 mm, still attached via frontal filament. Cephalothorax as in female. Genital complex about wider than long (0.63 x 0.52 mm), measured along the mid-line, excluding posterolateral lobes; with more or less parallel lateral margins and very conspicuous posterolateral lobes (Fig. 8B). Abdomen 2-segmented; first segment much shorter than wide (0.09 mm x 0.31 mm), second segment 3.4 times longer than first and wider than long (032 x 0.30 mm); carrying paired caudal rami distally; anal slit terminal. Caudal rami with parallel sides, just wider than long, measured at midpoints of margins. Each ramus armed with short hirsute seta at inner distal angle, slightly longer hirsute seta at outer distal angle, minute hirsute seta located just ventral to outer distal seta, and 3 setae on distal margin (2 long and plumose; middle seta reduced, non-plumose). Inner margin of ramus ornamented with setules as in male (Fig. 8B); single sensilla present on dorsal surface near inner distal corner.

Antennules, mandible, maxillule and maxilla as in female. Antenna modified (Fig. 8C); first segment elongate with single corrugated adhesion pad along posterior surface; second segment reflexed, elongate, bearing corrugated adhesion pads posteriorly, ventrally and anteriorly; distal segment forming strongly recurved simple claw, armed with 2 setae proximally (only 1 visible in figure). Post-oral process (Fig. 7D) better developed than in female and with corrugated surface.

Maxilliped (Fig. 8E) as for female except with rounded myxal process on proximal segment (syncoxa) opposing tip of claw of subchela, and with single large process proximally on posterior surface.

Sternal furca (Fig. 7D) with paired accessory processes located either side of furca, each with irregular lobulate surface, as in female.

Legs 1 to 4 as in female.

Leg 5 (Fig. 8B) forming extended tapering lobe at posterolateral corner of genital complex, bearing single (outer protopodal) seta laterally at base, plus 2 slender (exopodal) setae at apex. Leg 6 (Fig. 8B) represented by 2 setae on lobate distal corner of genital operculum.

*Remarks*: Despite the presence of a frontal filament the male specimen is clearly an adult male because it carries fully developed, corrugated adhesion pads on the antenna and these are secondary sexual characters which are only fully expressed at the final moult to adult as in *Caligus punctatus* Shiino, 1955 (see Kim, 1993 and Ho & Lin, 2004). Similarly, the male maxilliped, with its myxal process, also displays its secondary sexual form. In addition, fully formed, paired spermatophores are visible through the body wall of this male, indicating that it is a mature adult.

Conspecificity with the female, which was also still attached to the host by a frontal filament, is inferred from the shared multilobulate processes located either side of the sternal furca. Simple processes are present in this position in a few other species, such as *Caligus coryphaenae* (cf. Kabata, 1979) and *C. sicarius* Kabata, 1984 (Boxshall, 2018), but such multilobed processes are unique within the genus *Caligus* and serve to distinguish this species from all of its congeners. The conspecific female also seems to be adult, but is probably not yet mated. In this case the shape of the genital complex may not provide a clear indication of the typical shape of the individual adult female since genital complex shape can vary along with reproductive state.

**Genus:** ***Lepeophtheirus*** **von Nordmann, 1832**

Type species: *Lernaea pectoralis*, Müller, 1776, by original designation.

***Lepeophtheirus acutus*** **Heegaard, 1943**

*Host*: *Acroteriobatus annulatus* (Müller & Henle, 1841)

*Locality*: Muizenberg Beach (34°6′9.72″S, 18°29′23.75″E), collected on 16 April 2007

*Material examined*: 1 female deposited in the collections of the Natural History Museum (London) (NHMUK 2015.523).

*Description*: This species was redescribed in detail by Tang et al. (2013).

*Remarks*: This species was originally described based on material from *Taeniura lymma* (Forsskål, 1775) caught in the Western Pacific off the Gilbert Islands (Heegaard, 1943). It has subsequently been reported from a variety of rajiform, carcharhiniform and orectolobiform elasmobranchs held in captivity in aquaria (Kik et al., 2011) or in sea pens (Tang et al., 2013). These reports included a single record of *L. acutus* from a captive rhinobatid host, *Glaucostegus typus* (Anonymous [Bennett]) in Burger’s Zoo in The Netherlands. It has recently been reported from several hosts caught in the wild: *Aetobatus narinari* (Euphrasen) caught off Campeche, in the southern Gulf of Mexico (Rodriguez-Santiago et al., 2016), *Rhinobatos rhinobatos* (Linnaeus) and *Aetomylaeus bovinus* (Geoffroy Saint-Hilaire) caught in Turkish Mediterranean waters (Özak et al., 2018), and *Aetobatus ocellatus* (Kuhl) and *Himantura* cf. *astra* Last, Manjaji-Matsumoto & Pognoski caught in Moreton Bay, Queensland (Boxshall, 2018).

*Acroteriobatus annulatus* is a new host record and this is the first record of *L. acutus* from South African waters since the identity of the *Lepeophtheirus* sp. reported from *Rhinobatos* sp. by Barnard (1955) cannot be confirmed.

***Lepeophtheirus nordmanni*** **(Milne Edwards, 1840)**

*Host*: *Mola mola* (Linnaeus, 1758)

*Locality*: Table Bay (33°55′34.71″S, 18°22′18.87″E), collected on 20 October 2005 and 15 January 2008

*Material examined*: 12 females and 2 males. Vouchers: 8 females and 1 male in the Iziko South African Museum, (SAMC-A088689); 4 females and 1 male in the Natural History Museum, London (NHMUK 2014. 680-684).

*Representative DNA sequences*: GenBank: MW911363, MW925121

*Description*: Kabata (1979) described the key features of both sexes.

*Remarks*: According to Kabata (1979) this distinctive species has been recorded from *Mola mola* from both sides of the North Atlantic, the Mediterranean, the South Atlantic (Gulf of Guinea), the North Pacific (Japan and California), and off New Zealand. The record of *L. nordmanni* from *Thunnus* sp. (Oldewage, 1993) host is atypical and should be verified.

***Lepeophtheirus spinifer*** Kirtisinghe, 1937

*Syn. Dentigryps spinifer* (Kirtisinghe, 1937)

*Host*: *Rachycentron canadum* (Linnaeus, 1766)

*Locality*: Ushaka Sea World, Durban South Africa (29°52′4.53″S, 31° 2′44.30″E), collected on 02 July 2004

*Material examined*: 5 females and 2 males. Vouchers: 3 females and 1 male in the collections of the Iziko South African Museum (SAMC-A088690); 2 females and 1 male in the Natural History Museum, London (NHMUK 2014.705-706 and 2014.754).

*Representative DNA sequences*: GenBank: MW911364, MW925122

*Description*: Both sexes were redescribed and illustrated by Pillai (1985).

*Remarks*: This species was originally collected from a *Scomberoides* species (as *Chorinemus* sp.) caught off Sri Lanka (Kirtisinghe, 1937), and has subsequently been reported from India from *Rachycentron canadum* (Rangnekar, 1959), and from *Scomberoides lysan* (Forsskål) (as *Chorinemus lysan*) and *S. tala* (Cuvier) (as *Chorinemus tala)* (see Pillai, 1985). Lewis (1964) suggested a possible affinity between *L. spinifer* and the genus *Dentigryps* Wilson, 1913, and Ho & Dojiri (1977) subsequently transferred it to *Dentigryps*. However, *Dentigryps* is now accepted as a junior synonym of *Lepeophtheirus* (see Dojiri & Ho, 2013 for summary of history of this genus).

This species was not listed by Dippenaar (2005) in her overview of siphonostomatoid copepods reported from marine fishes of southern Africa and is a new record for South Africa.

Molecular analyses

Novel CO1 and 18S sequence data were generated for six of the 13 caligid species included here, namely *C. dakari*, *C. furcisetifer*, *C. lalandei*, *C. tetrodontis*, *L. nordmanni* and *L. spinifer* (GenBank accession numbers MW911361-MW911366 and MW925119-MW925124 as provided above). It was not possible to generate sequences for either gene for *A. gracilis*, *C. lineatus*, *C. longipedis*, *C. rufimaculatus, C. tenuis*, *C. tumulus* and *L. acutus* due to either specimen availability, or degradation and failure to amplify and/or sequence.

**Figure 9:**
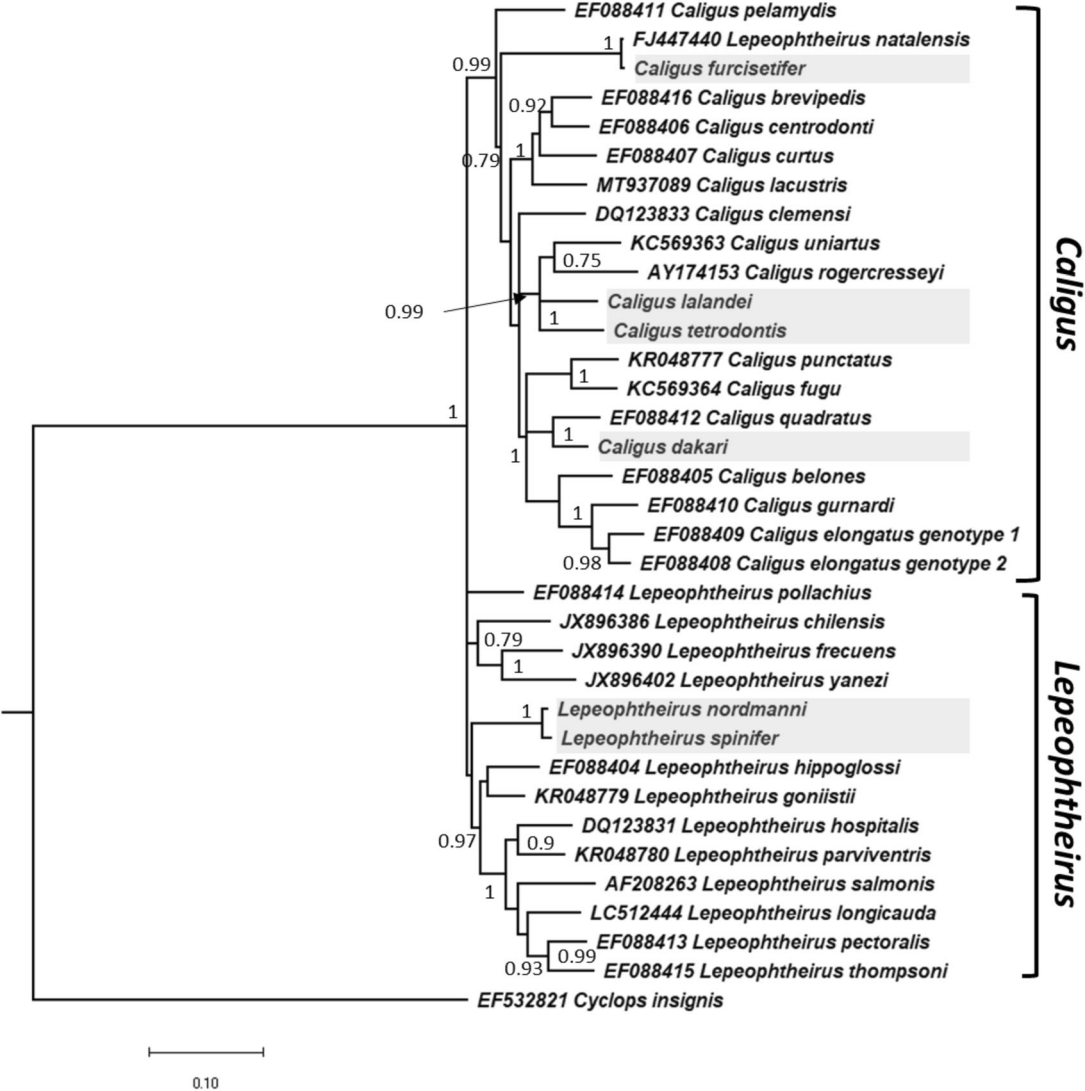
Bayesian inference analysis of the concatenated CO1 mtDNA and 18S rDNA dataset to highlight the phylogenetic positions of *Caligus* and *Lepeophtheirus* species from South Africa. Posterior probabilities are shown as nodal support, except for values below 0.7. The cyclopoid copepod *Cyclops insignis* Claus, 1857 was used as the outgroup.

Bayesian analysis produced a well-supported phylogeny with a distinct monophyletic *Caligus* clade and paraphyletic *Lepeophtheirus* grouping (Figure 9). Each of the South African species resolved as separate species within their respective genera, with two exceptions (see below). The South African *Caligus* did not cluster closely together within a single geographical specific subclade. *Caligus dakari* resolved as a sister taxa to *C. quadratus*, while C*. tetrodontis* and *C. lalandei* formed a distinct subclade with *C. rogercresseyi* and *C. uniartus. Caligus furcisetifer* and *L. natalensis* fell into a subclade together that appeared to be basal to the rest of the genus *Caligus*, with the exception of *Caligus pelamydis*. As mentioned above, based on this analysis and an uncorrected *p-*distance of 0.002 (0.2% divergence) *C. furcisetifer* and *L. natalensis* are indistinguishable as separate species based on the 3% divergence threshold for species delineation using DNA sequences (Herbert et al., 2003), which was supported by morphological comparison.

Unlike the *Caligus* species, the two South African *Lepeophtheirus* species, *L. nordmanni* and *L. spinifer*, did resolve as sister taxa forming a distinct subclade with the genus. However, the uncorrected *p-*distance between these two species was only 0.010 (1%) and application of the 3% divergence threshold for species delineation would suggest that these species are synonymous. However, there are numerous significant morphological differences between these species including body length (12 mm in female *L. nordmanni* compared to 4 mm in female *L. spinifer*) and the form of the female leg 5 (short and lobate in *L. nordmanni* compared to elongate and spiniform in *L. spinifer*). The relationship between these two species requires further investigation.

## Discussion

The position of *Lepeophtheirus natalensis* in molecular phylogenetic analyses of the caligids has been anomalous as it is recovered separate from other *Lepeophtheirus* species (Freeman et al., 2013). This has led to questioning of the monophyletic status of *Lepeophtheirus* (Morales-Serna et al., 2013). The discovery here that *L. natalensis* is a synonym of *Caligus furcisetifer* eliminates this conflict, although the status of *Lepeophtheirus* requires further testing with a larger taxon set including representatives of a greater diversity of caligid genera.

In order to facilitate identification within the species-rich *Caligus*, a number of species groups have been recognized. These informal groupings now accommodate just over half of the approximately 270 valid species currently contained in the genus. At present seven species-groups have been recognized, each based on the common possession of a suite of characters, but the phylogenetic status of these groups has not been tested (Boxshall, 2018; Hamdi et al. 2021). Neither of the new species can be placed in one of the seven recognized species-groups of *Caligus*.

The phylogenetic analysis undertaken here was not designed to test these species-groups so that, for example, *C. pelamydis* is the only representative of the *C. diaphanus*-group (see Boxshall, 2018) included in the analysis and, similarly, *C. dakari* is the only representative of the *C. productus*-group (see Boxshall & El-Rashidy, 2009). No representatives of the *C. bonito*-group (see Boxshall, 2018), the *C. confusus*-group (see Boxshall, 2018), the *C. pseudorhombi*-group (see Ohtsuka & Boxshall, 2019), or the *C. undulatus*-group (Ohtsuka *et al.*, 2020) were included. However, several species belonging to the *C. macarovi*-group, first proposed by Boxshall & Gurney (1980), are included in the taxon set.

The *C. macarovi*-group is characterized by the possession of a 3-segmented leg 4 with the first and second exopodal segments bearing I and III spines, respectively; the distal exopodal segment of leg 1 is armed with 3 posterior margin plumose setae and with spines 1, 2 and 3 all subequal in length, only spines 2 and 3 carry accessory processes, and seta 4 is markedly longer than spines; the proximal segment of the female antenna bears a posterior process; the distal margin of the brachium of the maxilla is typically ornamented with marginal serrations; and the abdomen is 1-segmented in the female. This group currently contains 44 species of which four, *C. lalandei*, *C. tetrodontis*, *C. rogercresseyi* Boxshall & Bravo, 2000 and *C. punctatus*, are listed as members of the *C. macarovi*-group by Boxshall (2018). The first three of these belong to a single clade (Fig. 9) which also contains *C. uniartus*, a species formerly placed in *Pseudocaligus* on the basis of its vestigial leg 4. Freeman et al. (2013) demonstrated that the reduction of leg 4 occurred several times within *Caligus* and that it is not a robust character at the genus level. We infer that *C. uniartus* may be closely related to the *C. macarovi*-group despite the reduced state of leg 4. On an adjacent branch in the tree (Fig. 9), *C. fugu* Yamaguti, 1936 is recovered as sister to *C. punctatus*. *Caligus fugu* is another former member of the invalid genus *Pseudocaligus* (characterized by a reduced leg 4) and may also be closely related to the *C. macarovi*-group. The tree morphology recovers the *C. macarovi*-group as paraphyletic but all these proposed groups need to be robustly tested with a much larger taxon set.

Prior to this study, the southern African caligid fauna comprised a total of 58 species accommodated in nine genera (Dippenaar, 2005). Here we increase that number with the addition of one species of *Alebion*, two species of *Lepeophtheirus* and four species of *Caligus*, two of which are new species. This constitutes the first record of *C. furcisetifer* from South Africa but this species has been previously reported in South African waters under the name of its junior synonym, *Lepeophtheirus natalensis*.
